# Temporal photoproximity labeling of ligand-activated EGFR neighborhoods using MultiMap

**DOI:** 10.1038/s41589-025-02076-y

**Published:** 2025-11-18

**Authors:** Zhi Lin, Wayne Ngo, Yu-Ting Chou, Harry Wu, Katherine J. Susa, Young-wook Jun, Trever G. Bivona, Jennifer A. Doudna, James A. Wells

**Affiliations:** 1 Department of Pharmaceutical Chemistry, University of California, San Francisco, San Francisco, CA USA; 2 Innovative Genomics Institute, University of California, Berkeley, Berkeley, CA USA; 3 Gladstone Institute of Data Science and Biotechnology, San Francisco, CA USA; 4 California Institute for Quantitative Biosciences, University of California, Berkeley, Berkeley, CA USA; 5 Department of Cellular and Molecular Pharmacology, University of California, San Francisco, San Francisco, CA USA; 6 Helen Diller Family Comprehensive Cancer Center, University of California, San Francisco, San Francisco, CA USA; 7 Department of Medicine, University of California, San Francisco, San Francisco, CA USA; 8 Department of Otolaryngology, University of California, San Francisco, San Francisco, CA USA; 9 Chan-Zuckerberg Biohub, San Francisco, CA USA; 10 Department of Molecular and Cell Biology, University of California, Berkeley, Berkeley, CA USA; 11 Gladstone-UCSF Institute of Genomic Immunology, San Francisco, CA USA; 12 Howard Hughes Medical Institute, University of California, Berkeley, Berkeley, CA USA; 13 Molecular Biophysics and Integrated Bioimaging Division, Lawrence Berkeley National Laboratory, Berkeley, CA USA; 14 Department of Chemistry, University of California, Berkeley, Berkeley, CA USA; 15 Li Ka Shing Center for Genomic Engineering, University of California, Berkeley, Berkeley, CA USA

**Keywords:** Target identification, Membrane proteins, Chemical tools, Proteomics

## Abstract

Photoproximity labeling proteomics (PLP) methods have recently shown that cell surface receptors can form lateral interactome networks. Here, we present a paired set of PLP workflows that dynamically track neighborhood changes for oncogenic epidermal growth factor receptor (EGFR) over time, both outside and inside of cells. We achieved this by augmenting the multiscale PLP workflow we call MultiMap, where three photoprobes with different labeling ranges were photoactivated by one photocatalyst, eosin Y, anchored extracellularly and intracellularly on EGFR. We identified hundreds of neighboring proteins that changed within minutes to over 1 h after the addition of EGF. These neighborhoods reveal dynamic interactomes during early, middle and late signaling that drive phosphorylation, internalization, degradation and transcriptional regulation. This rapid ‘molecular photographic’ labeling approach provides snapshots of signaling neighborhoods, revealing their dynamic nature and potential for drug targeting.

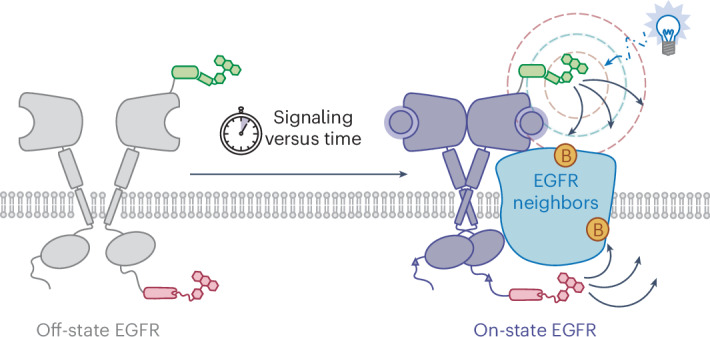

## Abstract (web summary)

A multiscale photoproximity labeling proteomics workflow captures dynamic neighborhoods of extracellular and intracellular epidermal growth factor (EGF) receptor interactomes during early, middle and late signaling upon activation by EGF.

## Main

Epidermal growth factor receptor (EGFR) is a classic paradigm for growth factor signaling^1^. As one of the first transmembrane receptors to be mechanistically and structurally characterized, EGFR activation is tied to multiple cytosolic signaling steps with different timescales^2–7^. EGFR is also a key therapeutic node in cancer signaling^8,9^. Antibody and small-molecule therapeutics targeting the extracellular domain (ECD) or kinase domain of EGFR have proven effective in delaying cancer progression^10–12^. However, cancers invariably develop resistance to these receptor-centric treatments and the presence of EGFR on normal tissues can create dose-limiting toxicity^13,14^. Thus, targeting functional EGFR interactors and other cancer-associated partners may provide an alternative strategy.

Proteins in the cell membrane are densely packed at distances estimated to be 60–70 Å apart, which is about the width of an average protein^15–17^. The two-dimensional environment in the membrane allows weak complexes to form rapidly and stably even when intrinsic dissociation constants can be 2–3 orders of magnitude lower than their three-dimensional counterparts^18^. These physical considerations have led to the development of in situ photoproximity labeling proteomics (PLP) using highly reactive carbene intermediates such as μMap^19–22^. In this method, an iridium photocatalyst conjugated to an antibody to trigger an aryl-diazirine-biotin photoprobe to form carbene species with an estimated labeling radius of ~110 Å from a single conjugation site on the primary antibody^23^. We recently reported the development of MultiMap^24^, a multiscale photocatalytic PLP platform that uses a single commercially available photocatalyst, eosin Y (EY), to trigger labeling at multiple ranges from ~100 to 3,000 Å (ref. ^25^) using three different photoprobes containing an aryl-diazirine, aryl-azide or phenol warhead^26–28^. Using MultiMap, we conjugated EY to cetuximab, a clinically approved EGFR antibody that competes with its natural ligand EGF and blocks EGFR signaling^29^, and probed this off-state neighborhood of EGFR with adjustable resolution. However, to map functional EGFR neighborhoods, we sought a universal way to label surface proteins with minimal impact on ligand stimulation events from both inside and outside the cells to allow for the study of dynamic interactions and signaling processes at multiple resolutions.

Here, we introduce a PLP approach based on MultiMap where one can systematically interrogate EGFR neighborhoods with enhanced coverage and precision by anchoring EY using genetically encoded epitope tags on the ECD or the intracellular domain (ICD). The full-length EGFR was engineered with an extracellular N-terminal Flag tag or an intracellular C-terminal HaloTag without interfering with signaling. The EY photocatalyst was attached using an EY-conjugated anti-Flag antibody for ECD ecto-tag MultiMap or EY–HaloTag ligand (EY–HTL) for ICD ecto-tag MultiMap. By adding EGF and triggering PLP reactions with blue light at different time points, we identified more than 300 proteins whose labeling patterns changed over a period of 5 min to 1 h. We validated ten candidates most highly associated with EGFR activation including phosphatases, trafficking proteins and transcription factors, reflecting early, middle and late signaling processes. This approach captures both temporally resolved and transient neighborhood changes of the EGFR signaling complexes that may inspire new targets for drug discovery.

## Results

### Genetically encoded tags for selective live cell labeling

**Fig. 1:**
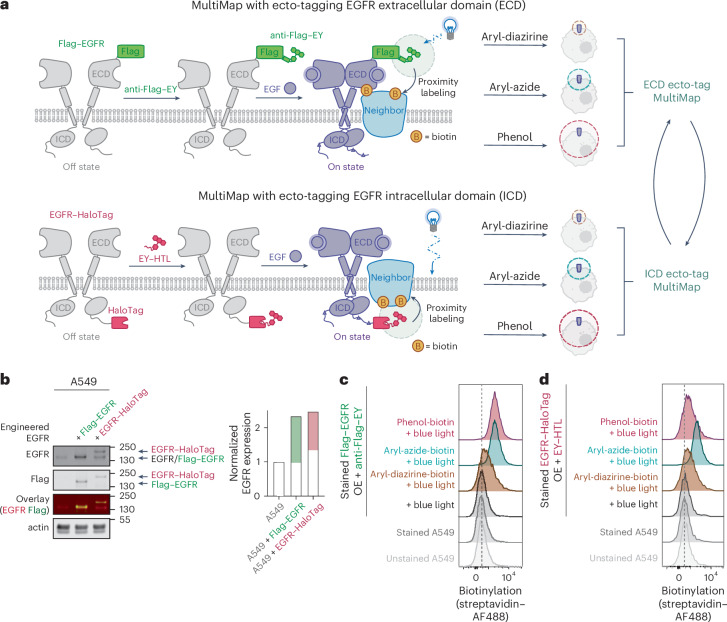
Systematic interactome profiling in live cells enabled by ECD and ICD ecto-tag MultiMap. **a**, Schematics of ecto-tag MultiMap workflows enabling photocatalytic biotinylation of neighboring proteins from either the ECD or the ICD of EGFR. The photocatalyst, EY, is introduced to EGFR by incubating cells expressing an N-terminal Flag tag (Flag–EGFR) or a C-terminal HaloTag (EGFR–HaloTag); EY is anchored using an EY-conjugated anti-Flag antibody or a derivatized EY with an HTL, respectively. Cells are illuminated for 2 min with blue light in the presence of three different biotinylated photoprobes of increasing labeling distances (aryl-diazirine-biotin, aryl-azide-biotin and phenol-biotin). EGFR and its neighbors are biotinylated, enriched and digested for MS analysis. These photoprobes allowed for tracking dynamic neighborhoods under different conditions, including EGF-triggered activation. **b**, For ECD and ICD ecto-tag MultiMap workflows, Flag–EGFR or EGFR–HaloTag were expressed in the cells at roughly equimolar amounts relative to endogenous EGFR as detected by western blot analysis. The small size difference between Flag–EGFR and endogenous EGFR cannot be resolved by western blot; EGFR–HaloTag is 33 kDa larger compared to endogenous EGFR, resulting in an upshifted band. **c**, Cellular labeling of A549 cells expressing Flag–EGFR with aryl-diazirine-biotin, aryl-azide-biotin and phenol-biotin in the presence of EY-conjugated Flag antibody (anti-Flag–EY) and blue-light illumination. **d**, Cellular labeling of A549 cells expressing EGFR–HaloTag with aryl-diazirine-biotin, aryl-azide-biotin and phenol-biotin in the presence of EY–HTL and blue-light illumination. Source Data

To label EGFR on either side of the membrane with the EY photocatalyst (Fig. 1a), we fused a Flag tag on the N terminus of the ECD of EGFR and a HaloTag on the C terminus of the ICD, designated Flag–EGFR and EGFR–HaloTag (Flag–EGFR–HaloTag), respectively (Supplementary Fig. 1a). We introduced these into A549 cells, a non-small cell lung cancer cell line, for its well-established and robust response to EGF activation and its physiologically relevant expression level of EGFR^30,31^. After transfecting the two constructs in A549 cells, we saw significant expression of the anti-Flag signal on cells for both constructs by flow cytometry (Supplementary Fig. 1b). We adjusted transfection conditions so that both engineered EGFR constructs, Flag–EGFR and EGFR–HaloTag, are at comparable levels to the endogenous EGFR (Fig. 1b).

To validate that the tagged EGFR constructs functioned properly upon EGF ligand activation, we incubated the engineered cells with EGF and monitored EGF-stimulated cellular behavior at 5 min, 10 min, 30 min and 60 min of activation in comparison to ligand-free conditions (Supplementary Fig. 1c). In particular, we observed significant activation of well-known and functional phosphorylation sites on EGFR (Y1086, Y1045 and Y1068)^32^ at comparable levels to the wild-type (WT) EGFR (Supplementary Fig. 1c,d). We also confirmed that cell engineering did not significantly alter the activation patterns of downstream signaling, including ERK phosphorylation, Akt phosphorylation, SHC phosphorylation, STAT5 phosphorylation and PLCγ phosphorylation upon EGF activation. In addition, these dynamic changes were continuous throughout the 5-min, 10-min, 30-min and 60-min intervals. We also performed an EGF on-cell binding assay and observed similar binding profiles comparing the engineered EGFR and endogenous EGFR (Supplementary Fig. 1e).

We then tested the Flag tag and HaloTag constructs for target-specific PLP. For the Flag tag strategy, we first transfected A549 cells with the Flag–EGFR construct and serum-starved the cells for >12 h to remove serum-induced EGF signaling. An EY-conjugated Flag antibody, prepared as previously reported^24^, was added to bind to the ECD-displayed Flag tag, followed by incubation with the three photoprobes for 15 min, namely aryl-diazirine-biotin, aryl-azide-biotin and phenol-biotin, to allow cell penetrance. Cells were then illuminated with blue light to activate labeling. As we aimed to obtain enhanced temporal resolution to track signaling events as rapid as 5 min, we optimized the light activation step and observed significant labeling with 2 min of blue-light activation on purified BSA and on live A549 cells (Supplementary Fig. 1f,g). We also confirmed that the labeling reactions with the photoprobes did not continue after 2 min of light activation when the samples were placed in the dark, suggesting that this reaction is light dependent and that the temporal resolution can be controlled by external light. By performing the full workflow on cells, we confirmed significant total cell labeling by flow cytometry using aryl-diazirine-biotin, aryl-azide-biotin and phenol-biotin with 2 min of blue-light activation (Fig. 1c and Supplementary Fig. 1h). We observed stronger labeling signals with the aryl-azide-biotin and phenol-biotin in comparison to aryl-diazirine-biotin, which is consistent with cell surface biotinylation from our previous studies. Cells were lysed and biotinylated proteins were purified on streptavidin beads, trypsinized and analyzed by mass spectrometry (MS). As expected, EGFR was one of the most enriched proteins with all three photoprobes (Supplementary Fig. 1h). When performing cellular localization analysis against established annotations, we observed a significant enrichment of both plasma membrane (PM) proteins and cell surface proteins (CSPs) (Supplementary Fig. 1i).

To anchor EY to the ICD of the engineered EGFR–HaloTag, we synthesized an EY hexyl-chloride derivative we call EY–HTL as previously reported (Supplementary Fig. 2a, Supplementary Note 2)^33^. Recent work has shown that conjugation using the HaloTag and HTL pair is highly efficient, selective, and compatible with cellular experiments^34–36^. Similar to the Flag-tagged ecto-tag MultiMap workflow, we incubated the A549 cells expressing EGFR–HaloTag with EY–HTL to allow selective and covalent anchoring of EGFR–HaloTag. To remove free EY–HTL from cells, we washed the cells four times with fresh medium, including 15-min soaks between washes (Supplementary Fig. 2a). The labeling and washout procedure was optimized using a well-established fluorophore with an HTL developed by the Lavis lab (JF646–HTL)^37^. Flow cytometry showed a dramatically increased fluorescent signal for the A549 cells expressing EGFR–HaloTag with 88% population shift compared to A549 parental cells, thus validating the washout procedure (Supplementary Fig. 2b).

To test the specificity of the EY–HTL on a non-interacting target using this procedure, we transfected GFP into A549 cells with and without HaloTag and incubated with EY–HTL (Supplementary Fig. 2c). We conducted labeling experiments followed by proteomics analysis and observed highly selective enrichment of GFP over any other native proteins with all three photoprobes (Supplementary Fig. 2d). We then performed the same workflow using EY–HTL on cells expressing EGFR–HaloTag and saw significant total cell labeling with all three photoprobes (Fig. 1d). In particular, while we continued to observe a similar degree of labeling with aryl-diazirine-biotin and aryl-azide-biotin, we observed a lower level of overall labeling with phenol-biotin; we hypothesized that the intracellular labeling environment may have quenched the phenol-biotin reaction. Thus, we collected a panel of quenching metabolite and enzymes commonly found in cells, including superoxide dismutase, glutathione, glutathione peroxidase and catalase (Supplementary Fig. 2e,f). We performed labeling with phenol-biotin on BSA and whole-cell lysate with different concentrations of these additives and found that the addition of glutathione significantly decreased phenol-biotin-based labeling in a dose-dependent manner, which supported that the intracellular components quench the labeling efficiency. Taken together, these results confirmed that target-specific cellular labeling was achievable with genetically encoded ECD Flag tag and ICD HaloTag and that both were compatible with the PLP workflow.

### EGFR neighborhood changes upon 5-min treatment with EGF

**Fig. 2:**
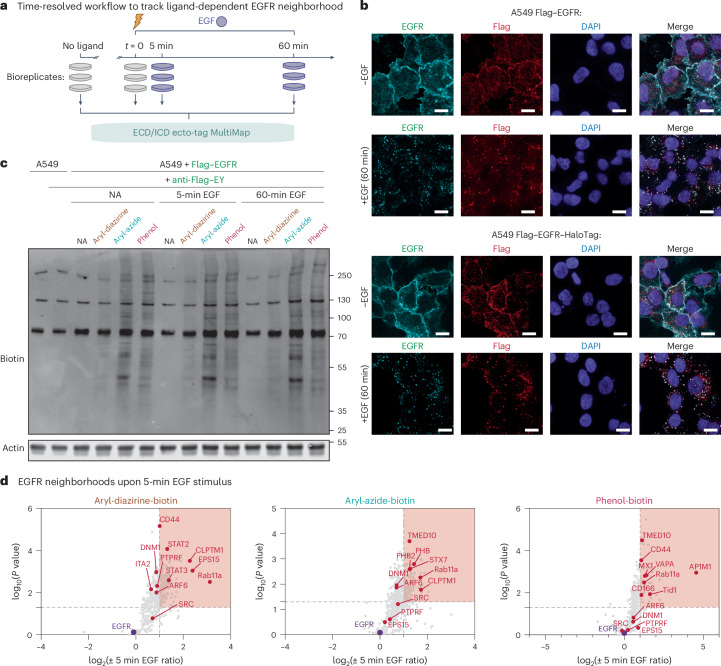
Rapid changes in EGFR neighborhoods upon EGF stimulation captured by time-resolved MultiMap. **a**, General time-resolved MultiMap workflows to monitor EGF-activated EGFR neighborhood changes. **b**, Confocal microscopy images of endogenous and Flag–EGFR (top) or EGFR–HaloTag (bottom) in cells treated with or without EGF. The staining of Flag–EGFR and EGFR–Halo overlapped with those from the endogenous EGFR, suggesting that trafficking is indistinguishable. Scale bar, 20 μm. **c**, Western blot analysis showing cellular biotinylation of A549 cells expressing Flag–EGFR using different photoprobes with short (5 min) and prolonged (60 min) EGF stimulation. **d**, Volcano plots of on-state EGFR neighborhoods comparing proximity labeling with aryl-diazirine-biotin, aryl-azide-biotin or phenol-biotin using ECD ecto-tag MultiMap after 5 min of stimulation with EGF versus no EGF. Significantly enriched proteins are highlighted in red (log_2_(fold enrichment) ≥ 1, *P* < 0.05, at least two unique peptides, three biological replicates) and *P* values were determined using Welch’s ANOVA. Data are tabulated in Supplementary Tables 7–9.

Signaling through EGFR is known to be transient with an activation stage followed by a deactivation and receptor turnover phase^6,7,38^. We sought to monitor the changes in EGFR neighborhoods upon ligand activation in a time-resolved manner. EGF-triggered EGFR processes such as phosphorylation, dephosphorylation and internalization occur rapidly within minutes to over 1 h. Short light exposure (2 min) triggered ample protein labeling (Supplementary Fig. 1f,g), thus making it possible to capture neighborhood changes with high temporal precision (Fig. 2a).

We further showed using confocal imaging that Flag–EGFR and EGFR–HaloTag traffic with similar kinetics and localization to endogenous EGFR before and after EGF activation (Fig. 2b and Supplementary Fig. 3a,b). All EGFR species (Flag–EGFR, EGFR–HaloTag and endogenous EGFR) were largely internalized within 60 min of EGF stimulation, with an overall Pearson correlation coefficient above 0.6. We performed the time-resolved ECD ecto-tag MultiMap workflow on cells in the presence of EGF (Fig. 2a). Through western blot analysis, we observed significant labeling on cells after EGF treatment for 5 min and 60 min, respectively (Fig. 2c).

We then conducted large-scale proteomics experiments with ECD ecto-tag MultiMap at different time points by capturing biotinylated proteins on streptavidin beads, followed by trypsinization and MS analysis. We found a large number of proteins enriched upon 5 min of EGF activation over the ligand-free state. After normalizing protein enrichment ratio to EGFR, we identified that 158 proteins increased in labeling with aryl-diazirine-biotin, 149 proteins increased in labeling with aryl-azide-biotin and 54 proteins increased in labeling with phenol-biotin using the same statistical threshold (log_2_(fold enrichment) ≥ 1, *P* < 0.05, at least two unique peptides, three biological replicates). The volcano plot in Fig. 2d, heat map in Supplementary Fig. 3c and Venn diagram in Supplementary Fig. 3d show overlap among the three probes. The cellular localization profiles are similar among probes, with more than 80% of the identified proteins classified as PM proteins or CSPs (Supplementary Fig. 3e). We also observed a significant fraction of proteins that were differential across the photoprobes as expected from our previous studies^24^. Many factors including different labeling efficiency (aryl-azide > phenol > aryl-diazirine), breadth of amino acid preference (aryl-diazirine > aryl-azide > phenol) and labeling radius driven by reactive half-life (phenol > aryl-azide > aryl-diazirine) along with detection threshold all contribute to the differences, suggesting that all probes are useful for a more holistic view of the neighborhood.

We identified 262 enriched proteins in total (Fig. 2d) with many top proteins of interest and annotated these proteins across volcano plots using different photoprobes. A number of neighbor candidates we identified were previously reported to directly interact with EGFR or functionally associate with EGFR. For example, the Ras-related protein, Rab11a, is reported to colocalize with EGFR upon EGF stimulation^39^ and Rab11a overexpression accelerates EGFR recycling in cells^40^. The EGFR pathway substrate, EPS15, was consistently found in our data and is reported to be associated with EGFR internalization and degradation^41,42^. We found ARF6, an ADP-ribosylation factor, to be highly enriched upon treatment with EGF. ARF6 is reported to interact with EGFR and is dependent on EGFR palmitoylation and ARF6 myristylation^43^. DNM1 was highly enriched and is known to regulate EGFR internalization as demonstrated in knockout experiments^44^. Other enriched targets with one or more photoprobes include proteins with known functional association such as the tyrosine kinase Src^45^, DnaJ homolog subfamily A member 3 (Tid1)^46^, the known transcription factor interactor STAT3 (refs. ^47,48^), the known extracellular stabilizer CD44 (refs. ^49,50^) and the protein tyrosine phosphatase transmembrane receptor (PTPRF)^51^. Several other proteins were also enriched with at least one photoprobe, including VAPA and TMED10 that were found in other interactomics studies^52^, CD166 that was reported to interact and mediate EGFR phosphorylation^53^ and several others less known to associate with EGFR in human cells such as PHB, CLPTM1, MX1, STX7 and AP1M1.

### Changes in EGFR neighborhoods over 60 min of EGF treatment

**Fig. 3:**
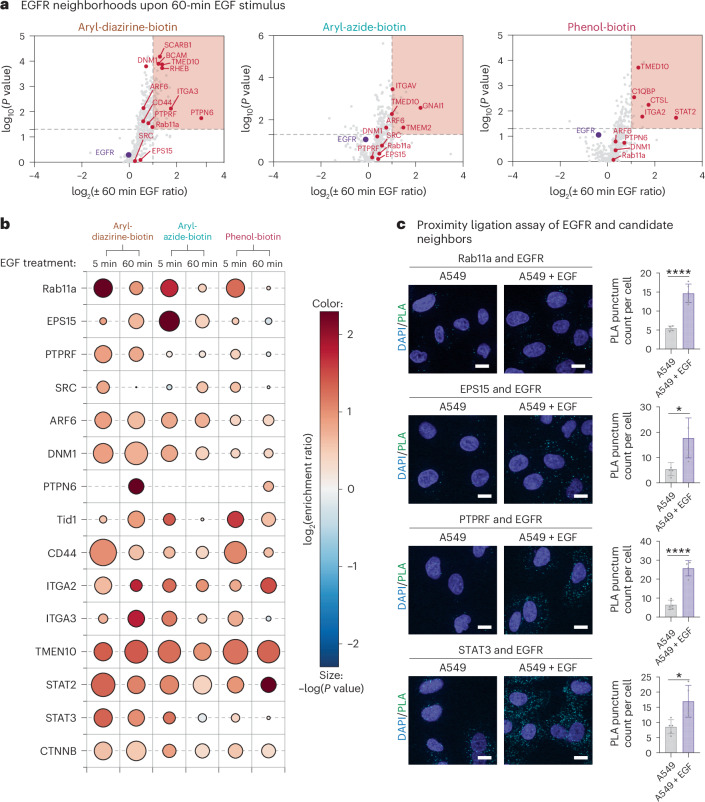
Time-resolved labeling by ECD ecto-tag MultiMap reveals changes in EGF-dependent EGFR neighbors viewed from outside. **a**, Volcano plots of on-state EGFR neighborhoods comparing proximity labeling with aryl-diazirine-biotin, aryl-azide-biotin or phenol-biotin using ECD ecto-tag MultiMap after 60 min of stimulation with EGF versus no EGF. Significantly enriched proteins are highlighted in the red box (log_2_(fold enrichment) ≥ 1, *P* < 0.05, at least two unique peptides, three biological replicates) and *P* values were determined using Welch’s ANOVA. Data are tabulated in Supplementary Tables 10–12. **b**, Enrichment profiles of candidate EGFR neighbors at 5 min and 60 min of EGF stimulation using the three photoprobes with different labeling ranges. The color (blue to red) represents increasing log_2_(fold enrichment) at indicated treatment times versus no EGF treatment. The size of bubbles indicates the −log(*P* value) across replicates calculated as described above, with larger sizes indicating higher confidence. **c**, PLA confirming colocalization of candidate EGFR neighbors with endogenous EGFR in an EGF-dependent manner. PLA assays were performed in nonengineered WT A549 cells after 5 min of EGF activation at a final concentration of 100 ng μl^−1^. Scale bar, 20 μm. Data are represented as the mean ± s.d. (*n* ≥ 4). *P* values were calculated using an unpaired two-way Student’s *t*-test, *P ≤ 0.05 and ****P ≤ 0.0001. Source Data

Next, we monitored the neighborhoods identified using ECD ecto-tag MultiMap over 60 min of EGF stimulation to evaluate whether the EGFR neighbors remain proximal to EGFR and to identify EGFR neighbors that enter later in the signaling (Fig. 3a). Significantly fewer proteins were enriched at the 60-min time point with all three probes: 76 proteins enriched with aryl-diazirine-biotin, 36 proteins enriched with aryl-azide-biotin and 24 proteins enriched with phenol-biotin using the same statistical threshold (log_2_(fold enrichment) ≥ 1, *P* < 0.05, at least two unique peptides, three biological replicates; volcano plot in Fig. 3a, heat map in Supplementary Fig. 4a, Venn diagram in Supplementary Fig. 4b and cellular localization in Supplementary Fig. 4c). Some proteins were found to be associated at both short and longer times but to varying degrees, along with proteins that were uniquely identified with 5 min or 60 min of activation (Fig. 3a and Supplementary Fig. 4d). In the Gene Ontology analysis, we found EGFR-associated molecular function terms such as protein binding, enzyme binding and protein-containing complex binding among the most enriched, together with biological process terms protein localization, cellular macromolecule localization and vesicle-mediated transport (Supplementary Fig. 4e).

We compared the time-dependent enrichment for the top protein hits that were highly enriched in one of the datasets or enriched throughout all datasets using a bubble plot (Fig. 3b). Rab11a, one of the most enriched proteins using all three photoprobes at 5 min of EGF stimulation, showed significantly lower enrichment ratios after 60 min of EGF stimulation. This suggests rapid association followed by disassociation and is consistent with the transient role of Rab11a in protein trafficking. Similarly, EPS15 was highly enriched at 5 min using aryl-azide-biotin but the enrichment ratio significantly dropped at 60 min.

Other proteins showed sustained levels of enrichment upon EGF stimulation such as PTPRF, Src, ARF6, DNM1 and CD44 at both short and long time points (Fig. 3b). Transcription factors such as STAT3, STAT2 and β-catenin also showed minor change in enrichment at 5 min and 60 min. Interestingly, enrichment ratios of several proteins became more prominent at 60 min compared to 5 min. This group of proteins included the protein tyrosine phosphatase nonreceptor type 6, PTPN6, also called SHP1, which was reported to physically interact with EGFR^54,55^, integrin A2 (ITGA2) and integrin AV, cathepsin L, which was characterized to mediate EGFR extracellular cleavage and shedding^56^, RHEB, which was recently reported to physically interact with EGFR when it enters the lysosome^57^, and other CSPs TMED10, SCARB1 and BCAM, which are not known to associate. It is likely that these proteins are recruited later to EGFR.

To validate these candidate neighbors and confirm that their engagement with EGFR is dependent on ligand activation, we sought to visualize them in situ using a proximity ligation assay (PLA) (Fig. 3c). PLA provides spatial proximity information with high sensitivity and is orthogonal to other assays such as coimmunoprecipitation^58,59^. We performed the PLA on five of the top hits with available and selective antibodies (Rab11a, EPS15, PTPRF, STAT3 and Src) in permeabilized WT A549 cells to visualize physical proximity of these proteins with EGFR in the presence of EGF (Fig. 3c and Supplementary Fig. 4f). When comparing ligand-free A549 cells and cells with EGF treatment, we found significant increases in PLA foci per cell upon EGF activation with Rab11a, EPS15, PTPRF and STAT3. Using flow cytometry as a detection method (Supplementary Fig. 4g), we observed the same pattern of EGF-dependent proximity to EGFR with larger cell populations, including EGFR neighbors Rab11a, EPS15, PTPRF, STAT3 and Src, as well as additional targets we identified in proteomics datasets, ARF6, DNM1 and β-catenin. While the PLA results cannot provide as high temporal resolution as PLP, PLA does provide orthogonal support for the physical proximity of these targets to EGFR upon EGF stimulation.

### ICD ecto-tag MultiMap maps intracellular neighborhoods

**Fig. 4:**
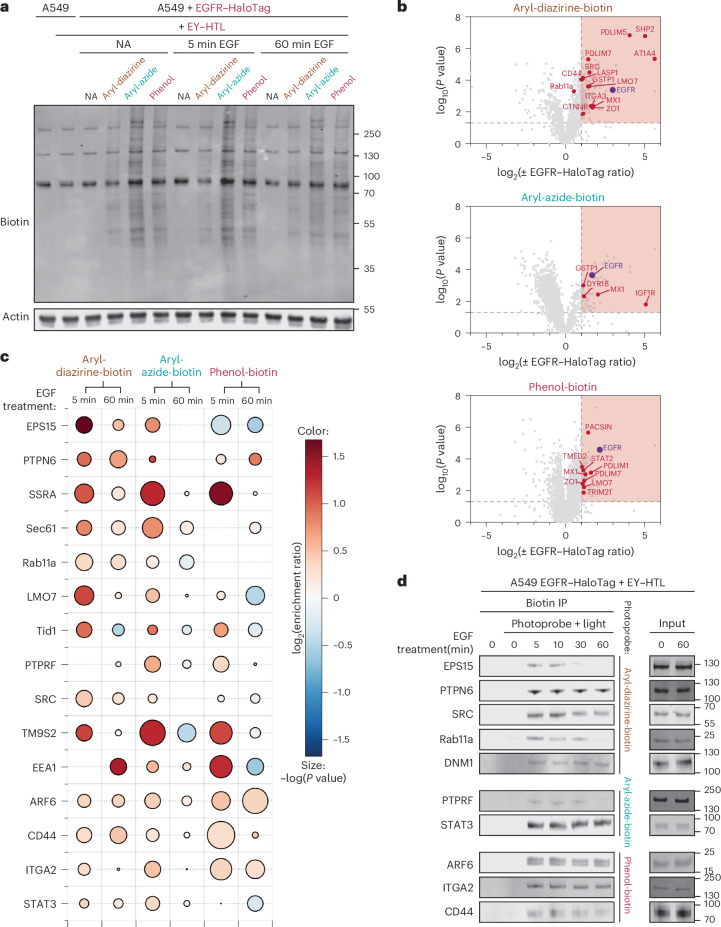
ICD ecto-tag MultiMap enabled identification and validation of neighbors of EGFR in an EGF-dependent manner viewed from inside. **a**, Cellular labeling of A549 cells expressing EGFR–HaloTag using different photoprobes with short (5 min) and prolonged (60 min) EGF stimulation. **b**, Volcano plots showing EGFR neighborhoods using ICD ecto-tag MultiMap. Labeled proteins using EY–HTL were compared with and without EGFR-HaloTag using the aryl-diazirine-biotin, aryl-azide-biotin and phenol-biotin photoprobes. Significantly enriched proteins are highlighted in red (log_2_(fold enrichment) ≥ 1, *P* < 0.05, at least two unique peptides, three biological replicates) and *P* values were determined using Welch’s ANOVA. Data are tabulated in Supplementary Tables 13–15. **c**, Enrichment profiles of candidate EGFR neighbors at 5 min and 60 min of EGF stimulation using the three photoprobes. The color (blue to red) represents increasing log_2_(fold enrichment) at the indicated treatment time versus no EGF treatment. The size of bubbles indicates the −log(*P* value) across three replicates calculated as described above, with larger sizes indicating higher confidence. **d**, Biotin immunoprecipitate (IP) western blots confirm that EGFR neighbors are captured in situ with EY–HTL-induced MultiMap at different time points.

Although the photoprobes used here are cell permeable in principle, we hypothesized that placing EY on the ICD of the EGFR could trigger greater proximity for identification of intracellular EGFR neighborhoods. We performed a similar PLP workflow where cells expressing EGFR–HaloTag were serum-starved and incubated with EY–HTL. We added repeated washout steps to remove noncovalently bound EY–HTL so as to reduce nonselective labeling (Supplementary Figs. 2 and 5a)^34–36,60^. EGF was added to cells followed by each of the three photoprobes and illuminated with blue light for 2 min to induce biotinylation (Supplementary Fig. 5a). We observed comparable bulk labeling in cells with no ligand, 5 min of EGF activation and 60 min of EGF activation by western blot analysis, reflecting effective biotinylation in all states (Fig. 4a).

We then used the ICD ecto-tag MultiMap workflow to identify the specific proteins enriched with and without EGFR–HaloTag (Fig. 4b and Supplementary Fig. 5b). EGFR was one of the most enriched proteins for all three probes in A549 cells. We also identified some EGFR neighbor proteins that we found with ECD ecto-tag MultiMap such as GSTP1, Src, Rab11a, ITA3, ZO1 and GSTP1, as well as other new proteins of interest including SHP2, which is a known positive regulator of EGFR signaling^61^, and several LIM domain proteins such as PDLIM5, PDLIM7, LMO7 and LASP1. We then compared proteins enriched upon EGF treatment at 5-min and 60-min time points (volcano plots in Supplementary Fig. 5c,d, heat map in Supplementary Fig. 6a, Venn diagram in Supplementary Fig. 6b and cellular localization in Supplementary Fig. 5c,d). We found a total of 105 proteins enriched with aryl-diazirine-biotin, 59 proteins enriched with aryl-azide-biotin and 229 proteins enriched with phenol-biotin at the 5-min EGF time point using standard metrics of log_2_(fold enrichment) ≥ 1, *P* < 0.05, at least two unique peptides and three biological replicates. At the 60-min time point, we found 112 proteins enriched with aryl-diazirine-biotin, 13 proteins with aryl-azide-biotin and 25 proteins with phenol-biotin using the same threshold. The ICD ecto-tag MultiMap data had significantly fewer enriched proteins annotated as PM proteins or CSPs at both 5 min and 60 min compared to those seen from our ECD ecto-tag MultiMap datasets (Supplementary Figs. 3e and 5c,d).

We performed comparative analysis of proteins identified at the 5-min and 60-min time points for ECD and ICD ecto-tag MultiMap datasets, respectively (Fig. 4b,c and Supplementary Figs. 5 and 6). The time-dependent enrichment profiles for individual proteins showed similarities, as visualized with bubble plots in Fig. 4c. For example, EPS15 was significantly more enriched at the 5-min time point compared to the 60-min time point; on the other hand, for CD44, ARF6 and STAT3, enrichment levels remained the same (Fig. 4c and Supplementary Fig. 5b,c). We also found some proteins with different enrichment profiles between ECD and ICD ecto-tag MultiMap datasets. For example, ICD ecto-tag enrichment data for Tid1 showed more significant changes at 5 min and 60 min than seen in the ECD ecto-tag data. Tid1 is an intracellular mitochondrial-associated protein that reportedly regulates the EGF-stimulated interaction with the Hsp70 chaperone system^46^. PTPN6, on the other hand, was similarly more enriched at 60 min by ICD ecto-tag using phenol-biotin, possibly for the same reason.

ICD ecto-tag MultiMap additionally provided new EGFR neighbor candidates (Fig. 4c and Supplementary Fig. 5b,c). This list includes the translocon-associated protein SSRA and Sec61 that are known to form complexes that facilitate the translocation of proteins including EGFR into the nucleus^62^, early endosomal proteins EEA1 and VPS that may engage with EGFR during endocytosis^63^, Rab27A that has important roles in cellular transport and TM9S2, a less studied protein.

We further validated the enriched hits seen in ICD ecto-tag MultiMap using western blots (Fig. 4d). Western blots depend on the quality of the antibodies available and may not have the sensitivity of MS. Nonetheless, we confirmed the increase in biotinylation of Rab11a, EPS15, Src, PTPN6 and DNM1 with aryl-diazirine-biotin enrichment, PTPRF and STAT3 with aryl-azide-biotin enrichment and ARF6, ITGA2 and CD44 with phenol-biotin enrichment at the 5-min time point, providing orthogonal support for direct association of neighboring proteins with EGFR. We also found a monotonic trend of enrichment levels from 5 min to 10 min, 30 min and 60 min of EGF activation. The combination of PLP and western blot analysis provides mutually supportive evidence that the identified EGFR neighbors are in physical proximity.

We also compared the neighborhoods identified by ECD and ICD ecto-tagged MultiMap (Supplementary Fig. 6e). They differ substantially, with less than 10% overlap of proteins identified. We believe that this reflects triggering of photoproximity labeling externally versus from within and highlights the complementary nature of these two methods. We also compared our datasets to published EGFR and STS1 interactome datasets that used AP-MS pulldowns and APEX2 in cells other than A549 cells (Supplementary Fig. 6f)^64,65^. While there were some common proteins, we were not surprised by the significant differences, likely because of different cell lines, treatment conditions, different experimental workflows and instruments used.

### EGFR neighbors are associated with EGF-activated functions

**Fig. 5:**
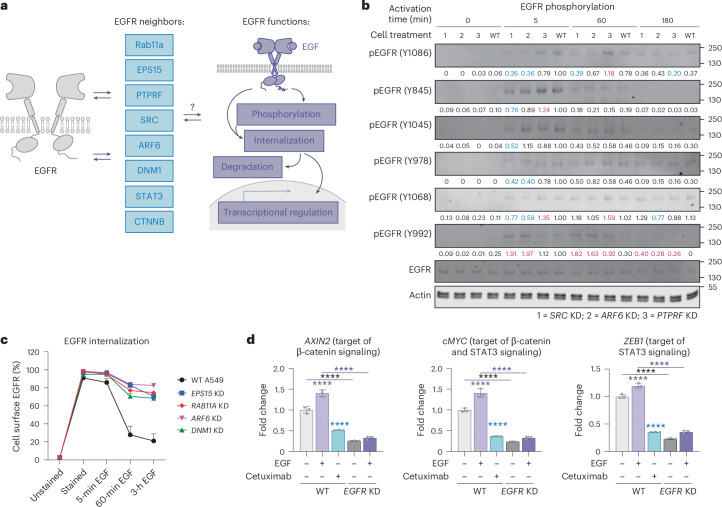
Ligand-activated EGFR neighborhoods dynamically regulate its signaling cascade. **a**, Summary of dynamically associating EGFR neighbors that change most with time as viewed by ECD and ICD ecto-tag MultiMap. **b**, Phosphorylation of EGFR at Y1086, Y845, Y1045, Y978, Y1068 and Y992 at different EGF activation time points in A549 cells with and without neighbor gene knockdowns. KD, knockdown. Normalized quantification results are shown beneath each band of interest. Elevated values are shown in red and decreased values are shown in blue. **c**, EGFR internalization in A549 cells at different EGF activation time points monitored with and without neighbor gene knockdowns. Data are quantified on the basis of the flow cytometry results of >10,000 cells, as shown in Supplementary Fig. 7b,c, with two bioreplicates. **d**, EGFR regulation on transcriptional activities of STAT3 and β-catenin measured by RT–qPCR analysis. Conditions with *EGFR* knockdown, EGF activation and EGF inhibition with cetuximab were compared. Data are represented as the mean ± s.d. (*n* = 3). *P* values were calculated using an unpaired two-way Student’s *t*-test. ****P ≤ 0.0001. Source Data

EGFR is known to undergo staged cytosolic signaling events over 1 h or so after EGF ligand activation, including phosphorylation, internalization, degradation and transcriptional regulation^7,66^. We were curious whether targets we identified by ECD and ICD ecto-tag MultiMap were regulating these processes. To begin to study this, we designed knockdown strategies against the most prominent candidate EGFR neighbors Src, ARF6, PTPRF, EPS15, Rab11a, DNM1, STAT3 and CTNNB from our datasets using CRISPR-mediated and small interfering RNA-based genetic perturbation and individually knocked down *SRC*, *ARF6*, *PTPRF*, *EPS15*, *RAB11A*, *DNM1* and *EGFR*, in A549 cells (Fig. 5). Western blot analysis confirmed selective knockdown of each target to over 50%, with the exception of *SRC*; the *SRC* gene is known to be essential and, thus, we limited it to 30% knockdown (Supplementary Fig. 7a). The gene knockdowns did not alter the overall levels of EGFR expression, suggesting that they did not perturb EGFR expression or trafficking.

Phosphorylation at tyrosine sites, including Y1086, Y845, Y1045, Y978, Y1068 and Y992, is known to be an immediate and critical regulatory mechanism behind EGFR signaling cascades (Fig. 5b and Supplementary Fig. 7a)^38,67^. We tested the differential kinetic patterns of phosphorylation at the different sites in WT A549 cells. As expected, we observed a significant increase in phosphorylation at all sites at 5 min of EGF stimulation, followed by dephosphorylation responses at Y845, Y1045 and Y992 and sustained phosphorylation at Y1086 and Y1068 sites at by 60 min. Knockdowns of *SRC* or *ARF6* significantly lowered the levels of EGFR phosphorylation at Y1086 and Y1068, which are the major autophosphorylation sites involved in the MAPK signaling cascade. Additionally, phosphorylation at the Y978 site decreased with *SRC* and *ARF6* knockdowns. *SRC* knockdown also had a mild influence on phosphorylation at the Y845 and Y1045 sites. Interestingly, knockdown of the gene for phosphatase PTPRF, a known tumor suppressor^68^, led to increased early phosphorylation of Y845 and Y1068 and late phosphorylation of Y1086 and Y992, whereas Y1045 and Y978 were largely unaffected.

We next sought to track time-dependent EGFR internalization by monitoring cell surface EGFR by flow cytometry at different time points of EGF activation at 37 °C (Fig. 5c and Supplementary Fig. 7a–c). With WT A549 cells, EGFR was internalized rapidly starting from 5 min of EGF activation and was kept internalized at the 60-min and 3-h time points (Supplementary Fig. 7b). After 3 h of EGF stimulation, we observed >60% EGFR internalization in WT A549 cells; knockdown of *RAB11A*, *EPS15*, *DNM1* or *ARF6* significantly reduced internalization of EGFR to only ~20% (Fig. 5c and Supplementary Fig. 7b,c). These data suggest that Rab11a, EPS15, DNM1 and ARF6 contribute to EGFR internalization upon EGF activation.

After internalization, EGFR can be degraded by 16 h, which is known to regulate EGFR activity. Knockdowns of *EPS15* and *DNM1* (Supplementary Fig. 7a,d) caused a significant reduction in EGFR degradation compared to WT cells in the presence of cycloheximide, suggesting that they may be involved in lysosomal trafficking (Supplementary Fig. 7d). Interestingly, knockdowns of *RAB11A* and *ARF6* had no significant effect at the 1-h and 3-h time points but slightly enhanced EGFR degradation at the 16-h time point. This may be contributed by Rab11a’s ability to mediate EGFR recycling from endosome back to membrane^39,69^. ARF6 may additionally regulate EGFR level by engaging the EGFR sorting process from Golgi to membrane^43^. These data support the roles of these proteins in promoting EGFR trafficking, which ultimately leads to EGFR turnover and homeostasis.

**Fig. 6:**
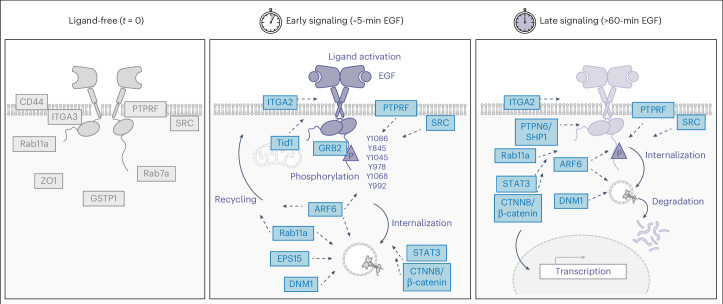
Model of EGFR association with functional neighbors identified using temporally resolved MultiMap. We mapped the dynamic neighborhoods of EGFR upon ligand activation using ECD and ICD ecto-tag MultiMap, which uncovered the time-dependent association of these functional neighbors with critical EGFR signaling processes including phosphorylation, internalization, degradation and transcriptional activation.

Activated EGFR also drives transcription through the regulation of a number of transcription factors including STAT3 and β-catenin^47,48,70,71^. As expected, both STAT3 and β-catenin were enriched through the ECD and ICD ecto-tag MultiMap upon EGF stimulation, indicating that they are in physical proximity (Figs. 3b and 4c). To further confirm the regulatory function of EGFR in the A549 cells, we conducted *EGFR* knockdown and monitored STAT3 and β-catenin transcriptional activities using qPCR analysis (Fig. 5d and Supplementary Fig. 7a). When WT A549 cells were stimulated with EGF, we observed increased transcriptional activities of STAT3 and β-catenin, leading to increased levels of the β-catenin-specific target gene *AXIN2*, the STAT3-specific target gene *ZEB1* and the shared β-catenin and STAT3 target gene *cMYC*^72,73^. On the other hand, EGFR inhibition with cetuximab reduced the gene expression for both STAT3 and β-catenin systems. When *EGFR* was knocked down, RNA levels of all these genes were significantly reduced and minimal induction was observed in the presence of EGF, which phenocopied the pattern with EGFR inhibition. These data support the hypothesis that transcriptional activities of both STAT3 and β-catenin are simultaneously modulated by EGFR. Collectively, these functional validation studies show that knockdown of candidate neighbors can affect EGFR signaling phenotypes including phosphorylation, internalization, degradation and transcriptional regulation (Fig. 6).

## Discussion

We present new temporally resolved MultiMap workflows, ECD and ICD ecto-tag MultiMap, that enable temporal tracking of protein neighborhoods during EGF-induced signaling processes. We and others have previously demonstrated labeling strategies to attach the photocatalyst using a selective antibody to the protein of interest (POI), such as cetuximab^24^, or receptor-specific ligands and protein-binding small molecules^74–79^. These approaches use epitopes on the POI with high target selectivity and can also block potential neighbors. By appending genetically encoded ecto-tags on both the inside and the outside of EGFR, we were able to introduce the EY photocatalyst outside the receptor coding sequence, which reduces the likelihood of blocking potential neighborhood interactions. Moreover, the ecto-tagging strategy does not require specific antibodies to the POI, thus greatly expanding the targetable proteome for photocatalytic PLP and integrating the advantage of being genetically encodable^80,81^. We did not observe any detectable effects on EGFR functions with these ecto-tags, and it is prudent to verify target protein functions in cells when applying to new protein targets.

ECD and ICD ecto-tag MultiMap allowed visualizing EGFR neighborhoods from both the ECD and the ICD. The two orthogonal methods provide a more comprehensive view, as evidenced by the fact that the two methods provided distinct enrichment profiles. We expected this difference in labeling for several reasons. Firstly, distance between the N-terminal Flag tag and and C-terminal HaloTag spans about 50 nm when one considers the length of EGFR (~40 nm) and the additional size of the anti-Flag antibody (150 kDa, ~10 nm) and the HaloTag (30 kDa, ~3 nm). This highlights the advantage of using multiple labeling probes that can cover possible distances. Secondly, we found that intracellular labeling using the phenol-biotin photoprobe was significantly restricted compared to extracellular labeling because of quenching by glutathione and, to a lesser extent, by enzymes such as superoxide dismutase and catalase. Lastly, the dense composition of proteins, lipids and other biomolecules on the membrane can provide a barrier for reactive species generated from photoprobes to pass from either direction. The 70-Å lipid bilayer alone is rich in C–H bonds that are known to react with carbene and nitrene intermediates^82,83^. Further studies on the cell permeability and local concentrations of both photocatalysts and probes can be beneficial. All these data highlight that the combination of ECD and ICD ecto-tag MultiMap methods provides a more holistic view of EGFR neighborhoods both on the cell membrane and as it traffics internally.

It is known that EGFR activation goes through a series of time-staged signaling processes ranging from the first minute to hours or days. These start with rapid phosphorylation and dephosphorylation, internalization or translocation, as well as prolonged regulation of the transcriptional activity and finally degradation. The short 2-min illumination for ECD and ICD ecto-tag MultiMap is well suited to probe these dynamic neighborhoods. This may be applicable to other proteins or proteoforms during their active signaling, including aberrant mutants^84,85^, drug mechanisms^86,87^ and cell-specific or organelle-specific neighborhoods^88,89^. Concurrent with the preparation of this manuscript, numerous innovative approaches have emerged in the proximity labeling field covering new photocatalysts^90–94^ or new mechanisms of activation^95–99^, along with remarkable advances at the frontier of applications^89,91,100–107^, highlighting the growing interest and applicability of the strategy. We believe that our complementary set of MultiMap tools expands the current strategies of μMAP, APEX, TurboID or BioID for dynamic neighborhood mapping during active signaling^35,77,108–113^.

The neighbors that we identified and confirmed by PLA and western blot analysis mostly had functional consequences when probed by gene knockdowns. We were able to systematically track functional neighbors in each process, including Src, ARF6 and PTPRF during their regulation of EGFR phosphorylation, Rab11a, EPS15, DNM1 and ARF6 when regulating EGFR internalization and STAT3 and β-catenin as downstream transcription factors, which provided a list of targetable entities (Fig. 6). PTPRF, PTPN6 and EPS15 are known to serve as tumor suppressors^68,114,115^, whereas Src kinase, Rab11a and CD44 may be tumor promoters^116–119^. We envision that this mechanistically detailed proximity map of EGFR functional neighborhood will help dissect the mechanisms behind these different processes and facilitate phenotype-specific probing of EGFR.

Taken together, the temporally resolved MultiMap workflows capture neighborhood snapshots with enhanced resolution, allowing insights to be gleaned holistically from different domains of the cell surface target. By dissecting the dynamic neighborhoods during its active signaling cascade, we can visualize the horizontal signaling of EGFR that occurs simultaneously upon vertical ligand activation. We believe that such studies will reveal new functional interactions and lead to new targets for therapeutic development.

## Methods

### General methods and instrumentation

Illumination was performed using a Penn PhD photoreactor M2 (Sigma-Aldrich, Z744035) with a 450-nm blue-light source module (Sigma-Aldrich, Z744033) at 100% intensity or light-emitting diode (LED) array light sources (Thor Labs, LIU470A for 470-nm LED array, LIU525B for 525-nm LED array) along with an LED mounting adapter (AD38). To use the Penn PhD photoreactor, fan speed was set at 6,800 rpm under manual control with 100 stirs per min and samples were illuminated at 100% intensity for the indicated time. To use LED array light sources, samples were placed under a Thor Labs LED array light source, which provides intensity of 4.0 mW cm^−2^ (470 nm) and 1.9 mW cm^−2^ (525 nm) at a distance of 100 mm from the LED according to information from the manufacturer. Flow cytometry experiments were performed on a CytoFlex flow cytometer (Beckman CytoFlex) and analyzed using FlowJo software. Proteomics experiments were performed on a TimsTOF PRO (Bruker) equipped with a CaptiveSpray source and a nanoElute System. The peptides were separated on a 25-cm ReproSil c18 1.5-μM 100-Å column (PepSep, PSC-25-150-15-UHP-nc). Protein quantification was performed using a bicinchoninic acid assay on a multimode microplate reader Infinite 200 PRO (Tecan Trading). Sonication of cells or protein pellets was performed using a QSonica Q500 Sonicator (QSonica Sonicators). DNA or protein concentrations were measured using a NanoDrop 2000 spectrophotometer (Thermo Scientific).

### Western blot protocol

For immunoblotting analysis, proteins were loaded on 4–12% 17-well Bis–Tris gels (Thermo Fisher, NW04127BOX), and transferred from SDS–PAGE gels to PVDF membranes (Thermo Fisher, IB24002) using an iBlot-2 dry blotting system (Thermo Scientific, IB21001). Membranes were blocked with Intercept (TBS) blocking buffer (LI-COR, 927-60001), incubated with the primary antibodies as indicated by the vendors, washed with Tris-buffered saline containing 0.1% Tween-20 (37 mM sodium chloride, 20 mM Tris, 2.7 mM potassium chloride and 0.05% Tween-20, pH 7.4) and incubated with secondary antibodies sequentially including anti-rabbit IgG goat IR800 secondary antibody (Rockland, 926-32211), anti-rabbit IgG Goat IR680 secondary antibody (Rockland, 611-144-002), anti-rabbit IgG Goat secondary antibody peroxidase (Rockland, 611-1302), anti-mouse IgG Goat IR800 secondary antibody (Rockland, 610-145-211) and/or anti-mouse IgG Goat IR680 secondary antibody (Rockland, 610-144-002). Immunoblots images were captured by an infrared LI-COR imager (Odyssey CLx). In-gel fluorescence and immunoblot fluorescence signals were detected on a Bio-Rad imager (ChemiDoc XRS+ system). Analysis was performed using ImageStudioLite.

### Cell culture and transfection

A549 cells were acquired from the University of California, San Francisco (UCSF) cell culture and banking services and cultured in DMEM (Thermo Fisher, 11995073), supplemented with penicillin (50 μg ml^−1^), streptomycin (50 μg ml^−1^) and 10% (v/v) FBS. Transfection was performed using TransIT-PRO (Mirus Bio, fMIR 5740) according to the manufacturer’s instructions.

### Antibodies and biological reagents

Antibodies were used at 1:1,000 dilution for western blot, 1:2,000 dilution for flow cytometry and 1:1,000 dilution for imaging staining unless otherwise noted (full lists in Supplementary Methods). Anti-M1-Flag monoclonal antibody was purified from M1 hybridoma. In brief, two 40-ml aliquots of supernatant isolated from M1 Flag clone 4E11 cells (American Type Culture Collection, CRL-3623)^120^ were diluted 1:1 with buffer containing 20 mM HEPES pH 7.4, 150 mM NaCl and 2 mM CaCl_2_. The diluted supernatant was loaded over a Flag peptide column and then washed with ten column volumes of cold washing buffer of 20 mM HEPES pH 7.4 with 150 mM NaCl and 2 mM CaCl_2_. Bound antibody was eluted with 2.5 column volumes of buffer containing 100 mM citrate pH 3.0 and the elution was immediately neutralized with 2 M HEPES pH 8.0. The purified antibody was dialyzed overnight into PBS and purity was assessed on a Coomassie-stained SDS–PAGE gel. Protein was concentrated to approximately 1 mg ml^−^^1^ and supplemented with 10% glycerol; small aliquots were flash-frozen in liquid nitrogen and stored at −80 °C until use.

### General flow cytometry

Cultured cells were incubated at 37 °C in 5% CO_2_ for the duration of the assay. To guarantee minimal cell activity, cells were moved onto ice right after treatment and washed three times with prechilled PBS. Whole-cell flow staining or cell surface staining were then performed on pelleted cells.

For cell surface staining, cells were blocked with filtered 3% BSA in PBS for 30 min at 4 °C and stained with corresponding antibody for 1 h at 4 °C. Stained cells were then washed three times with PBS and resuspended in PBS for flow cytometry analysis.

For whole-cell staining, cells were fixed with 4% formaldehyde for 10 min at room temperature, washed twice with PBS and then permeabilized with 100% prechilled methanol for 15 min at 4 °C. Cells were then washed before blocking with filtered 3% BSA in PBS for 30 min at 4 °C and stained with corresponding antibody for 1 h at 4 °C. Stained cells were then washed three times with PBS and resuspended in PBS for flow cytometry analysis. A minimum of 10,000 cells were collected for each sample. Flow cytometry data were analyzed on FlowJo. Live versus dead cells were gated first used FSC/SSC gating. Single cells were then gated by removing doublets in FSC-H/FSC-A gating.

### Flag-tagged labeling assay using anti-Flag antibody

Flag-based labeling was performed as indicated in the workflow in Supplementary Fig. 1h. In brief, transfected A549 cells were incubated at 37 °C in 5% CO_2_ to 80% confluency and serum-starved overnight. They were then washed with prechilled PBS three times. Indicated amounts of anti-Flag antibodies or antibody–EY conjugates were prechilled and added to the cells for 15 min at 4 °C before excessive antibody–EY conjugates were removed by washing with prechilled PBS. The antibody-bound cells were then resuspended in serum-free DMEM and treated as indicated. Cells were then moved to 4 °C before collection, resuspended in prechilled PBS supplemented with indicated photoprobe reagents (aryl-diazirine-biotin, aryl-azide-biotin or biotin-phenol) at a final concentration of 100 μM and left to incubate for 15 min at 4 °C. Thoroughly mixed cells were illuminated with the LED for the indicated time at 4 °C, pelleted and subjected to flow cytometry or liquid chromatography (LC)–MS/MS sample preparation.

### HaloTagged labeling assay using EY–HTL ligand

HaloTag-based labeling was performed as indicated in the workflows in Supplementary Fig. 5a. In brief, transfected A549 cells were incubated at 37 °C in 5% CO_2_ to 80% confluency and serum-starved overnight. They were then washed with PBS three times and incubated with indicated concentrations of EY–HTL for 30 min at 37 °C. Then, the excessive molecules were washed off with serum-free medium twice and incubated with serum-free medium for 15 min at 37 °C, which was repeated four times. EY–HTL-bound cells were then resuspended in serum-free DMEM and treated as indicated. Cells were then moved to 4 °C before collection, resuspended in prechilled PBS supplemented with indicated photoprobe reagents (aryl-diazirine-biotin, aryl-azide-biotin or biotin-phenol) at a final concentration of 100 μM and left to incubate for 15 min at 4 °C. Thoroughly mixed cells were illuminated with the LED for the indicated time at 4 °C, pelleted and subjected to flow cytometry or LC–MS/MS sample preparation.

### Biotinylated protein enrichment

To enrich biotinylated proteins, cell pellets were resuspended in 1 ml of 1× RIPA lysis buffer (EMD Millipore) supplemented with protease inhibitor (protease inhibitor cocktail 100×; Cell Signaling Technology, 5871). After 15 min of incubation on ice, cells were sonicated for 15 s (5 s on, 5 s off, 20%). Cell lysates were then cleared by centrifugation at 20,000*g* for 10 min at 4 °C. Proteins were then added to 150 μl of Pierce streptavidin agarose beads (Pierce, 20349) that were prewashed with 1 ml of PBS three times and incubated for 16 h at 4 °C. Afterward, the supernatant was discarded using mini Bio-spin columns (Bio-Rad) and the beads were washed three times with 1 ml of 1× RIPA lysis buffer, three times with 1 ml of 1 M NaCl in 1× PBS and three times with 1 ml of freshly prepared 2 M urea in 50 mM ammonium bicarbonate. The beads were then subjected to western blotting analysis or LC–MS/MS analysis.

### Sample preparation for LC–MS/MS analysis

Proteins on the washed beads were then digested using the Preomics iST kit in an on-bead digestion format according to the manufacturer’s instructions. In brief, washed beads were suspended in 100 μl of LYSE buffer provided by the Preomics iST kit and incubated at 55 °C for 10 min for reduction and alkylation. Once the beads cooled to room temperature, 50 μl of prereconstituted DIGEST was added to the beads and incubated at 37 °C for 3 h with shaking. The digested peptides were then collected using mini Bio-Spin columns (Bio-Rad) and another 50 μl of LYSE buffer was added to wash the beads. Afterward, 100 μl of STOP solution was added to the combined flowthrough elution and mixed using vigorous vortexing. Then, the peptides were desalted using the Preomics desalting columns before they were dried under vacuum and resuspended in 15 μl of solvent A (0.1% formic acid with 2% acetonitrile) for MS analysis. The peptide amount was monitored using a Pierce fluorometric peptide assay kit (Pierce, 23290) and a NanoDrop 2000 spectrophotometer (Thermo Scientific).

### Proteomics analysis of digested peptide samples

Proteomics experiments were performed on a TimsTOF PRO (Bruker) equipped with a CaptiveSpray source and a nanoElute system. The peptides were separated on a 25-cm, ReproSil c18 1.5-μm 100-Å column (PepSep, PSC-25-150-15-UHP-nc) using a stepwise linear gradient method with water in 0.1% formic acid (solvent A) and acetonitrile with 0.1% formic acid (solvent B): 5–30% solvent B for 90 min at 0.5 μl min^−1^, 30–35% solvent B for 10 min at 0.6 μl min^−1^, 35–95% solvent B for 4 min at 0.5 μl min^−1^ and 95% hold for 4 min at 0.5 μl min^−1^). Acquired data were collected in data-dependent acquisition mode with ion mobility activated in parallel accumulation–serial fragmentation mode. MS and MS/MS spectra were collected with *m*/*z* ranging from 100 to 1,700 in positive mode.

### Analysis of proteomics dataset

All acquired data were searched using PEAKS online Xpro 1.6 (Bioinformatics Solutions) or FragPipe powered by MSFragger (version 3.7). Spectral searches were performed using a curated FASTA-formatted dataset containing a Swiss UniProt-reviewed human proteome file with gene ontology localized on the PM (downloaded from UniProt database) or Swiss UniProt-reviewed human proteome. The precursor mass error tolerance was set to 20 ppm and fragment mass error tolerance was set to 0.03 ppm. Peptides, ranging from 6 to 45 aa in length, were searched in semi-specific trypsin digest mode with a maximum of three missed cleavages. Carbamidomethylation (+57.0214 Da) on cysteines was set as a static modification while methionine oxidation (+15.9949 Da) and lysine acetylation (+42.0115 Da) were set as variable modifications. Peptides were filtered on the basis of a false discovery rate of 1%. Samples were normalized using total ion current or EGFR. For *P*-value calculations, Welch’s analysis of variance (ANOVA) was implemented in PEAKS DB software.

### EGFR localization imaging analysis

EGFR localization analysis was performed using A549 cells plated on an 18-well chamber slide precoated with ibiTreat (iBidi, 81816) that were serum-starved overnight before treatment and with or without EGF for indicated time. Cells were then fixed with 4% paraformaldehyde freshly diluted from 16% paraformaldehyde (Electron Microscopy Sciences, 15710) for 10 min at 25 °C and permeabilized with 0.1% Triton in PBS for 15 min at 25 °C before blocking with 3% goat serum albumin (GSA) for 1 h at 4 °C. Primary antibodies diluted in 3% GSA were incubated at 4 °C overnight, followed by washes and incubation with diluted secondary antibodies for 1 h at 4 °C. Cells were finally stained with DAPI before confocal imaging. Confocal microscopy was performed using a ×60 Plan-Apo 679 (1.42 numerical aperture) oil objective on an Olympus Fluoview 3000 laser scanning confocal microscope. Images were acquired as Z-stacks using Galvano scanning mode and confocal zoom of ×5 magnification. Individual cells were manually segmented and colocalization was quantified with the Pearson correlation coefficient using the Coloc2 plugin in ImageJ.

### PLA using imaging as the detection method

The PLA was performed according to published literature^58,59^. In brief, A549 cells were plated on an 18-well chamber slide precoated with ibiTreat (iBidi, 81816) and serum-starved overnight before treatment with or without EGF for indicated time. Cells were then fixed with 4% paraformaldehyde freshly diluted from 16% paraformaldehyde (Electron Microscopy Sciences, 15710) for 10 min at 25 °C and permeabilized with 0.1% Triton in PBS for 15 min at 25 °C before a Duolink in situ PLA assay kit (Sigma-Aldrich, DUO92014-30RXN) was used according to the manufacturer’s instructions. In brief, the cells were blocked with 1× Duolink blocking buffer for 60 min at 37 °C before primary antibodies diluted with Duolink antibody diluent as indicated by antibody vendors were added. After incubation at 4 °C overnight, cells were washed with Duolink wash buffer A (Sigma-Aldrich, DUO82049-4L) and diluted PLA secondary antibodies (anti-mouse minus, DUO92004-30RXN; anti-rabbit plus, DUO92002-30RXN) were added and incubated for 60 min at 37 °C. After washes with Duolink wash buffer A, a ligation reaction was performed with supplied reagents from the Duolink in situ green detection kit (Sigma-Aldrich, DUO92014-30RXN) for 30 min at 37 °C, followed by an amplification reaction for 100 min at 37 °C. Cells were then washed with Duolink wash buffer B (Sigma-Aldrich, DUO82049-4L) and 100-fold diluted Duolink wash buffer B before the samples were stained with Duolink mounting solution with DAPI (Sigma-Aldrich, DUO82040-5ML) for 15 min in dark. Images were obtained on an inverted Nikon Ti-E microscope equipped with a Yokogawa CSU-22 confocal scanner unit. PLA spots in cells were counted automatically using imageJ QuickPALM function of analyzing particles, with the minimum signal-to-noise ratio set to 20.00 and maximum full-width at half maximum set as 7 px. Quantification was demonstrated as the average ± s.d. from at least four individual images. Image acquisition parameters and data processing parameters were the same for all treatment conditions.

### PLA using flow cytometry as the detection method

The PLA was performed using Duolink flowPLA detection kit—green (Sigma-Aldrich, DUO94002) according to the manufacturer’s instructions. In brief, A549 cells were plated on a tissue-culture-treated 10-cm plate and serum-starved overnight before treatment with or without EGF for indicated time. Cells were then moved onto ice, harvested and fixed with 4% paraformaldehyde freshly diluted from 16% paraformaldehyde (Electron Microscopy Sciences, 15710) for 10 min at 25 °C. The cells were permeabilized with 0.1% Triton in PBS for 15 min at 25 °C before a Duolink flowPLA detection kit (Sigma-Aldrich, DUO94002) was used. The cells were blocked with 1× Duolink blocking buffer for 60 min at 37 °C before primary antibodies diluted with Duolink antibody diluent as indicated by antibody vendors were added. After incubation at 4 °C overnight, cells were washed with Duolink wash buffer A (Sigma-Aldrich, DUO82049-4L) and diluted PLA secondary antibodies (anti-mouse minus, DUO92004-30RXN; anti-rabbit plus, DUO92002-30RXN) were added and incubated for 60 min at 37 °C. After washes with Duolink wash buffer A, a ligation reaction was performed with supplied reagents from the Duolink flowPLA detection kit for 30 min at 37 °C, followed by an amplification reaction for 100 min at 37 °C. Cells were then washed twice with Duolink wash buffer A before the samples were stained with Duolink detection buffer for 30 min at 37 °C in the dark. Stained cells were then washed twice with PBS and resuspended in PBS for flow cytometry analysis. A minimum of 10,000 cells were collected for each sample. Flow cytometry data were analyzed on FlowJo. Live versus dead cells were gated first used FSC/SSC gating. Single cells were then gated by removing doublets in FSC-H/FSC-A gating. The mean fluorescence intensity was calculated on single cells.

### RNA isolation and reverse transcription (RT)–qPCR experiment

Total RNA was extracted from cells with indicated treatment using RNeasy mini kit (Qiagen, 74106), QIAshredder (Qiagen, 79656) and RNase-free DNase set (Qiagen, 79254). RT was performed using Superscript II RTase (Thermo Fisher Scientific, 18064014). qPCR was carried out using the TaqMan fast advanced master mix (Applied Biosystems, 44-445-57) on a QuantStudio 12K Flex real-time qPCR system (Applied Biosystems). Target gene expression was measured with specific TaqMan probes for *AXIN2* (Hs00610344_m1), ZEB1(Hs01566408_m1), *cMYC* (Hs00153408_m1) and *GAPDH* (Hs02786624_g1). Relative gene expression levels were calculated using the ΔΔ*C*_t_ method and normalized to the housekeeping gene *GAPDH*.

### Software

Data were analyzed and visualized using GraphPad Prism (version 8.0.1) and Microsoft Excel (version 16.22), in addition to software listed by each experiment. NMR data were analyzed using MestReNova (version 15.0.0). DNA and protein sequences were analyzed using SnapGene (version 6.0.2). Flow cytometry data were analyzed using FlowJo (version 10.6.1). Proteomics data were analyzed using PEAKS online (Xpro version 1.6) and FragPipe powered by MSFragger (version 3.7). Images were made using ImageStudioLite (version 5.2.5), Adobe Illustrator (version 22.1) and BioRender (version 2.0).

### Statistics and Reproducibility

All data were derived from at least two biological replicate experiments. Statistical analyses (unpaired Student’s *t*-tests) were performed using GraphPad Prism. Data were presented as the mean ± s.d. (*P ≤ 0.05, **P ≤ 0.01, ***P ≤ 0.001 and ****P ≤ 0.0001; NS, not significant).

### Reporting summary

Further information on research design is available in the Nature Portfolio Reporting Summary linked to this article.

## Online content

Any methods, additional references, Nature Portfolio reporting summaries, source data, extended data, supplementary information, acknowledgements, peer review information; details of author contributions and competing interests; and statements of data and code availability are available at 10.1038/s41589-025-02076-y.

## Supplementary information

Supplementary Information Supplementary Figs. 1–7, Methods and Notes 1 and 2.

Reporting Summary

Supplementary Tables 1–24 Supplementary tables.

## Source data

Source Data Figs. 1, 3 and 5 Numerical source data.

## Data Availability

All experimental data, materials and methods are available in the main text or the Supplementary Information. The MS proteomics data were deposited to the ProteomeXchange Consortium through the PRIDE partner repository with the dataset identifier PXD059180. Source data are provided with this paper.
