# Supplementary material for: Temporal photoproximity labeling of ligand-activated EGFR neighborhoods using MultiMap
Source: Nat Chem Biol. 2025 Nov 18;22(2):192–204. doi: 10.1038/s41589-025-02076-y (PMC12858401; doi:10.1038/s41589-025-02076-y)
Supplement: Supplementary file 1 — Supplementary Figs. 1–7, Methods and Notes 1 and 2. [file 41589_2025_2076_MOESM1_ESM.pdf]

nature chemical biology

Supplementary information

https://doi.org/10.1038/s41589-025-02076-y

Temporal photoproximity labeling of ligand-activated EGFR neighborhoods using MultiMap

In the format provided by the authors and unedited

Supplementary Information:

Contents

Supplementary Figures:
Supplementary Fig. 1 | Design of ecto-tag MultiMap allowed for selective protein labeling.
Supplementary Fig. 2 | ICD ecto-tag MultiMap demonstrated high labeling selectivity in cells.
Supplementary Fig. 3 | Time-resolved changes in the EGFR neighborhoods captured by ECD ecto-tag MultiMap.
Supplementary Fig. 4 | ECD ecto-tag MultiMap can capture dynamic EGFR neighborhoods at different timepoints.
Supplementary Fig. 5 | Time-resolved ICD ecto-tag MultiMap neighborhoods facilitated by HaloTagged EGFR.
Supplementary Fig. 6 | Different and shared features identified via ECD and ICD ecto-tag MultiMap.
Supplementary Fig. 7 | Genetic manipulation of target genes revealed EGFR ligand-dependent functional neighbors.

Supplementary Methods:
General methods and instrumentation.
Complete list of antibodies and biological reagents.
Construct information and cell expression.

Supplementary Note
Supplementary Note 1
Supplementary Note 2

Supplementary Fig. 1 | Design of ecto-tag MultiMap allowed for selective protein labeling. **A,** Designs of EGFR constructs used in this study. **B,** Engineered EGFR constructs, Flag-EGFR and EGFR-HaloTag, were expressed and displayed on the cell surface of A549 cells. Both Flag-EGFR and EGFR-HaloTag contain ecto-displayed Flag-tags for flow cytometry staining. **C,** Engineered EGFR constructs respond indistinguishably to EGF stimuli (100 ng/μL) as seen in EGFR phosphorylation and downstream signaling activation. **D,** Quantification of EGF-induced activation of engineered EGFR species and endogenous EGFR at different phospho-sites showed similar activation patterns. **E,** Quantification of EGF-binding assays demonstrated similar engagement of engineered EGFR species and endogenous EGFR. **F,** Optimization of blue light activation on purified bovine serum albumin (BSA). **G,** Optimization of blue light activation on live A549 cells. **H,** Optimized workflow of ECD ecto-tag MultiMap using an anti-Flag-EY. **I,** Volcano plots of EGFR neighborhoods using ECD ecto-tag MultiMap. Labeled proteins in cells expressing Flag-EGFR using anti-Flag-EY were compared with those enriched in WT A549 cells using aryl-diazirine-biotin, aryl-azide-biotin, phenol-biotin, respectively. Significantly enriched proteins are highlighted in the red box [log2(fold enrichment) ≥ 1, P < 0.05, at least 2 unique peptides, three biological replicates] and P-values were determined using Welch's ANOVA. Data is tabulated in **Supplementary Table 1-3**. All annotations were exported from UniProt or previous literature. SURFY=predicted surfaceome in silico[1] (total protein number=2886, **Supplementary Table 22**); PM=plasma membrane (total protein number=5488, **Supplementary Table 23**); CSP=cell surface proteome[2] (total protein number=8212, **Supplementary Table 24**).

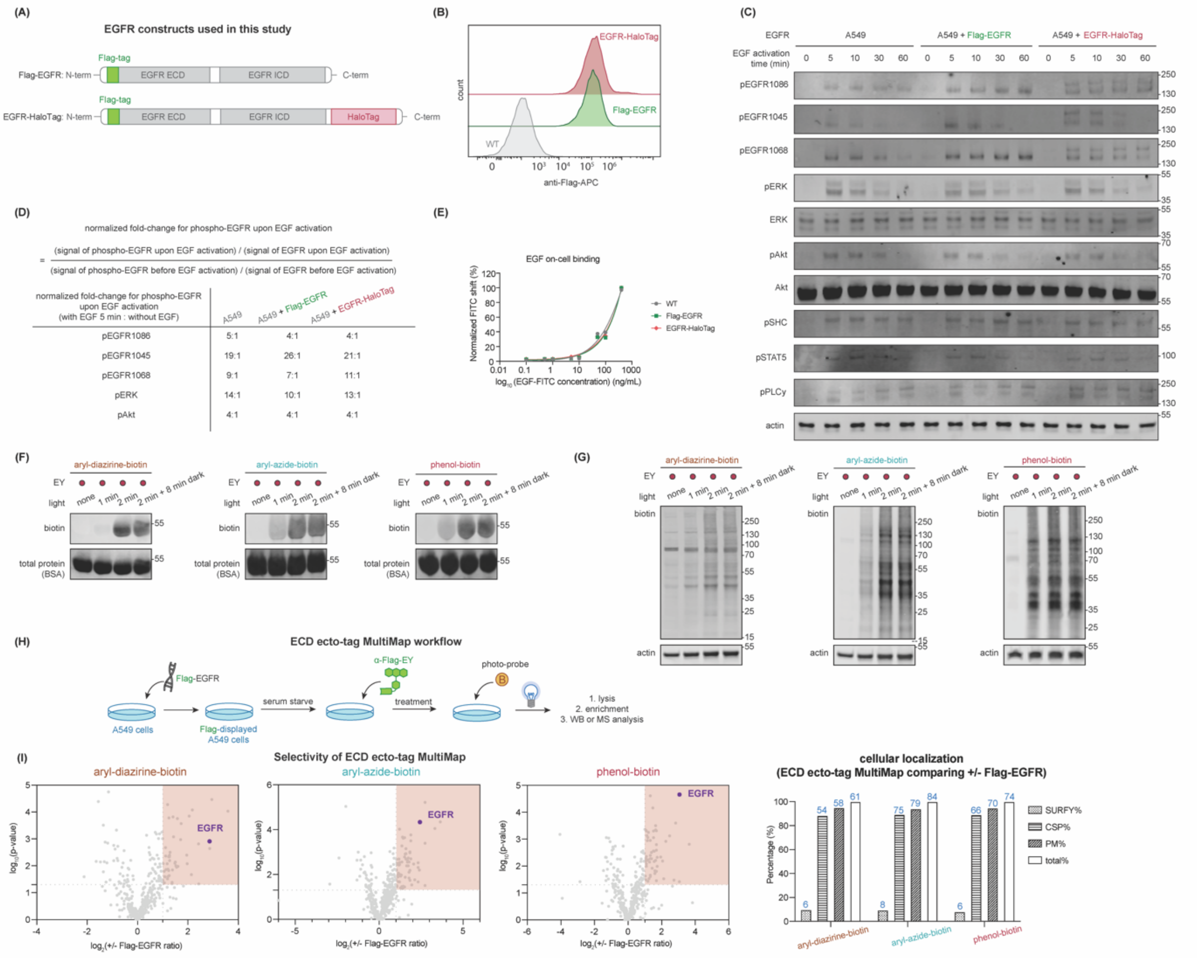

Supplementary Fig. 2 | ICD ecto-tag MultiMap demonstrated high labeling selectivity in cells. A, Chemical structure of EY-HTL and design of ICD ecto-tag MultiMap workflow that used 4 repeated cell washout steps to remove unbound EY-HTL. **B,** Flow cytometry diagram that validated selective incorporation of dye-HTL (JF646-HTL) with the washout steps. When incubated with JF646-HTL, A549 cells expressing EGFR-HaloTag (middle) retained a significant level of JF646-HTL signal after the washout steps in comparison to WT-A549 cells without HaloTag expression (up). **C,** Optimized workflow of ICD ecto-tag MultiMap using a GFP-HaloTag construct to validate the workflow. Cells expressing either GFP, or EGFP-HaloTag were incubated with EY-HTL, washed to remove unbound EY-HTL followed by photocatalytic proximity labeling proteomics analysis. **D,** Volcano plots of EY-HTL-enriched proteins in cells expressing GFP-HaloTag compared to those in cells expressing GFP without HaloTag using aryl-diazirine-biotin, aryl-azide-biotin, and phenol-biotin, respectively. Significantly enriched proteins are highlighted in the green box [log2(fold enrichment) ≥ 1, P < 0.05, at least 2 unique peptides, three biological replicates] and P-values were determined using Welch's ANOVA. Data is tabulated in **Supplementary Tables 4-6**. **E,** Characterization of effect of reactive oxygen processing enzymes (superoxide dismutase-1, glutathione peroxidase, and catalase) and the metabolite, glutathione, on phenol-biotin labeling on BSA. **F,** Characterization of effect of the same set of enzymes and metabolite on phenol-biotin labeling in A549 cell lysate.

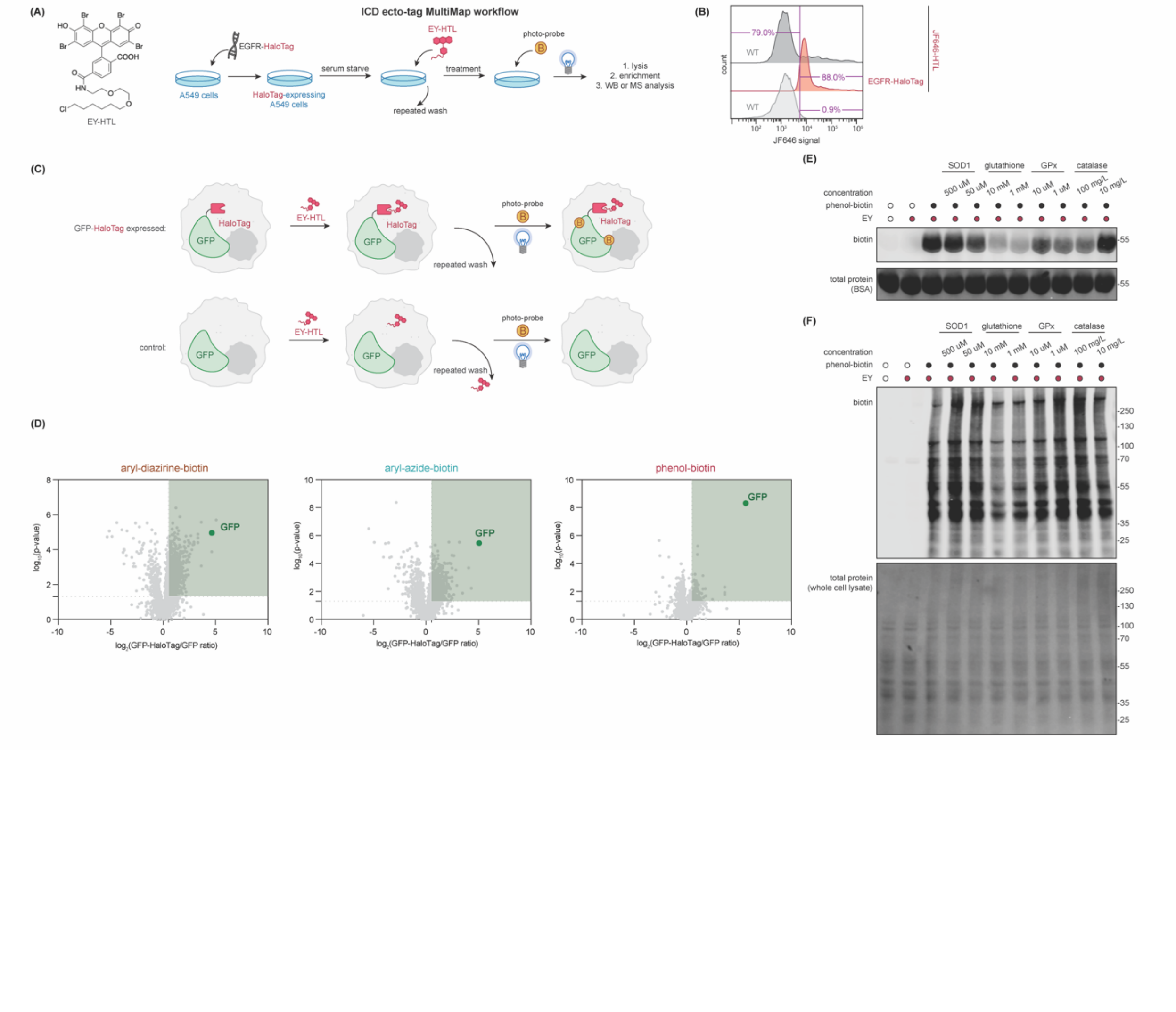

Supplementary Fig. 3 | Time-resolved changes in the EGFR neighborhoods captured by ECD ecto-tag MultiMap. A, Confocal microscopy images of endogenous EGFR in WT A549 cells treated with or without EGF for 60 min. Scale bar = 20 µm. **B,** Confocal microscopy images of total EGFR and transfected Flag-EGFR (top) and EGFR-HaloTag (bottom) in A549 cells with 5 min, 60 min EGF or without EGF treatment, demonstrating that both constructs are highly co-localized with endogenous EGFR before and after ligand activation. Experiments were described in **Fig. 2b** and data are represented as mean ± SD (n=10). **C,** Heatmap of the proteins identified in **Fig. 2d**. Color indicates enrichment ratio of 5 min EGFR neighborhood over ligand-free conditions, with red indicating increased enrichment and blue indicating decreased enrichment. **D,** Venn diagram of enriched EGFR interactome with 5 min incubation of EGF enriched from A549 cells by using three different photo-probes. **E,** Cellular localization of protein hits. All annotations were exported as described above.

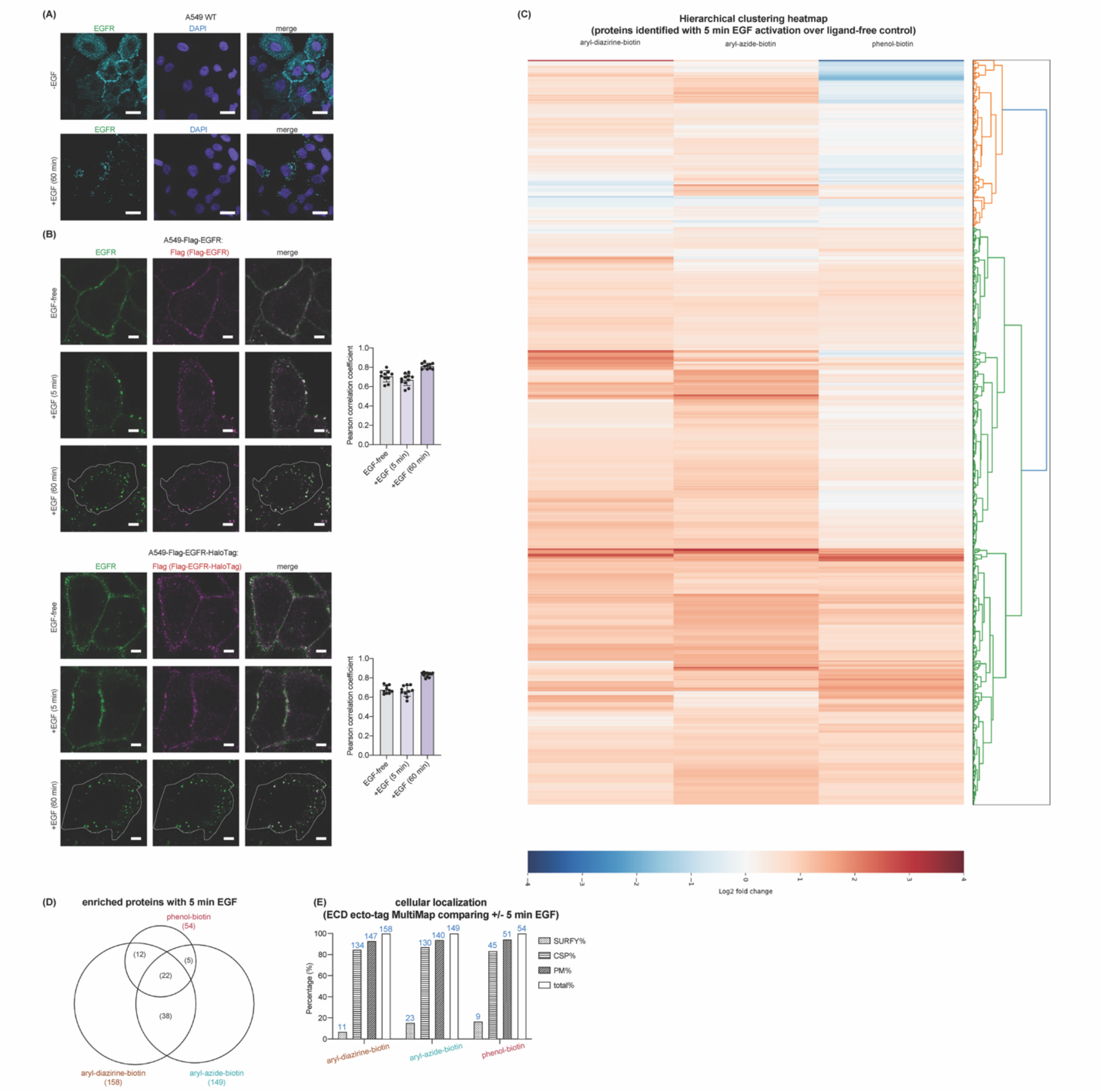

Supplementary Fig. 4 | ECD ecto-tag MultiMap can capture dynamic EGFR neighborhoods at different timepoints. A, Heatmap of the proteins identified in **Fig. 3a** compared to that from **Fig. 2d**. Color indicates enrichment ratio of treatment over ligand-free conditions, with red indicating increased enrichment and blue indicating decreased enrichment. **B,** Venn diagram of enriched EGFR interactome with 60 min incubation of EGF enriched from A549 cells by using different photo-probes. **C,** Cellular localization of protein hits analyzed similarly to **Supplementary Fig. 3e**. **D,** Direct comparison of enriched EGFR interactome captured with 5 min EGF and that with 60 min EGF. All annotations were exported as described above. **E,** Gene Ontology (GO) analysis performed on all EGFR-interacting candidates in **Fig. 2d**, **3a.** Candidates were compared to UniProt-reviewed human proteome file (downloaded from UniProt database). Top 20 most significant biological process terms and molecular function terms were annotated with -log10(P-value) with P-value calculated using Fisher's exact test with False Discovery Rate correction. **F,** Representative images of proximity ligation assay (PLA) to visualize proximity of EGFR neighbors, Rab11a, EPS15, PTPRF, STAT3 and SRC identified in **Fig. 3c**. PLA puncta are in green, indicating proximity between target and EGFR, and nuclei are in blue. Scale bar = 20 µm. **G**, Flow cytometry detection of PLA signal in large cell populations to visualize proximity of EGFR neighbors, Rab11a, EPS15, PTPRF, STAT3, SRC, ARF6, DNM1 and CTNNB in an EGF-dependent manner. PLA assays were performed in non-engineered WT A549 cells after 5 min EGF activation at a final concentration of 100 ng/µL, similar to **Fig. 3c**, for each indicated EGFR neighbor and EGFR. Both PLA signal (FITC) shift and MFI ratio, which indicates normalized mean fluorescence intensity of EGF-treated sample vs. ligand-free sample, were calculated.

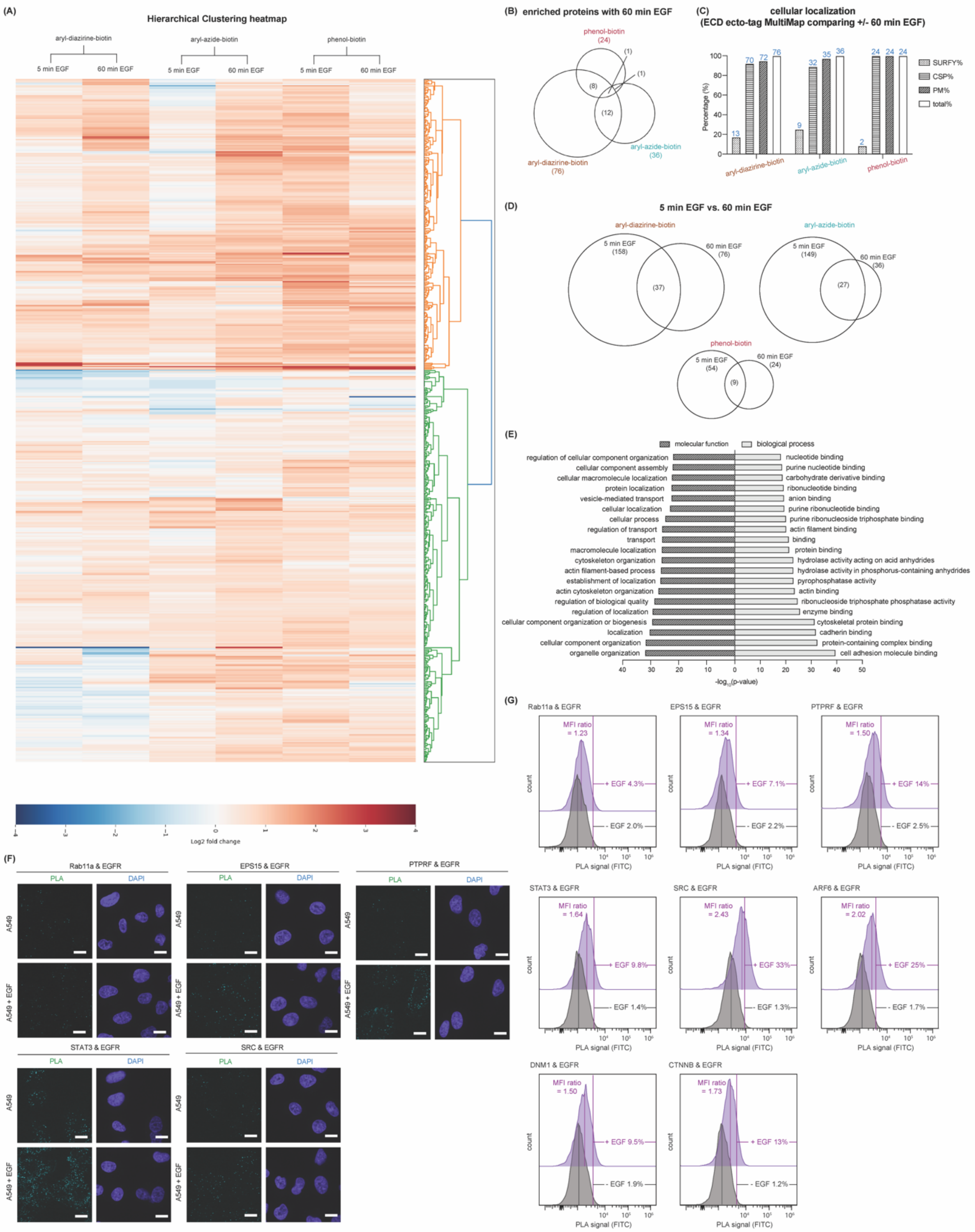

(A)
Hierarchical Clustering heatmap
aryl-diazirine-biotin
aryl-azide-biotin
phenol-biotin
5 min EGF
60 min EGF
Log2 fold change
(B) enriched proteins with 60 min EGF
phenol-biotin (24)
(1)
(1)
(8)
(12)
aryl-diazirine-biotin (76)
aryl-azide-biotin (36)
(C) cellular localization
(ECD ecto-tag MultiMap comparing +/- 60 min EGF)
Percentage (%)
SURFY%
CSP%
PM%
total%
13
70
72
76
9
32
35
36
2
24
24
24
aryl-diazirine-biotin
aryl-azide-biotin
phenol-biotin
(D)
5 min EGF vs. 60 min EGF
aryl-diazirine-biotin
5 min EGF (158)
60 min EGF (76)
(37)
aryl-azide-biotin
5 min EGF (149)
60 min EGF (36)
(27)
phenol-biotin
5 min EGF (54)
60 min EGF (24)
(9)
(E)
molecular function
biological process
regulation of cellular component organization
cellular component assembly
cellular macromolecule localization
protein localization
vesicle-mediated transport
cellular localization
cellular process
regulation of transport
transport
macromolecule localization
cytoskeleton organization
actin filament-based process
establishment of localization
actin cytoskeleton organization
regulation of biological quality
regulation of localization
cellular component organization or biogenesis
localization
cellular component organization
organelle organization
nucleotide binding
purine nucleotide binding
carbohydrate derivative binding
ribonucleotide binding
anion binding
purine ribonucleotide binding
purine ribonucleoside triphosphate binding
actin filament binding
binding
protein binding
hydrolase activity acting on acid anhydrides
hydrolase activity in phosphorus-containing anhydrides
pyrophosphatase activity
actin binding
ribonucleoside triphosphate phosphatase activity
enzyme binding
cytoskeletal protein binding
cadherin binding
protein-containing complex binding
cell adhesion molecule binding
-log10(p-value)
(F)
Rab11a & EGFR
EPS15 & EGFR
PTPRF & EGFR
STAT3 & EGFR
SRC & EGFR
PLA
DAPI
A549
A549 + EGF
(G)
Rab11a & EGFR
MFI ratio = 1.23
+ EGF 4.3%
- EGF 2.0%
EPS15 & EGFR
MFI ratio = 1.34
+ EGF 7.1%
- EGF 2.2%
PTPRF & EGFR
MFI ratio = 1.50
+ EGF 14%
- EGF 2.5%
STAT3 & EGFR
MFI ratio = 1.64
+ EGF 9.8%
- EGF 1.4%
SRC & EGFR
MFI ratio = 2.43
+ EGF 33%
- EGF 1.3%
ARF6 & EGFR
MFI ratio = 2.02
+ EGF 25%
- EGF 1.7%
DNM1 & EGFR
MFI ratio = 1.50
+ EGF 9.5%
- EGF 1.9%
CTNNB & EGFR
MFI ratio = 1.73
+ EGF 13%
- EGF 1.2%
count
PLA signal (FITC)

Supplementary Fig. 5 | Time-resolved ICD ecto-tag MultiMap neighborhoods facilitated by HaloTagged EGFR. A, We integrated the time-resolved ligand treatment workflow with ICD ecto-tag MultiMap, which enabled photocatalytic proximity labeling in cells. **B,** Cellular localization of protein hits. All annotations were exported as described above. **C,** Volcano plots for on-state EGFR neighborhoods that result after 5 min treatment with EGF versus no treatment using ICD ecto-tag MultiMap. After EGF treatment, cells were put on ice and illuminated to trigger labeling using either aryl-diazirine-biotin, aryl-azide-biotin, phenol-biotin photo-probes. Significantly enriched proteins are highlighted in the red box [log2(fold enrichment) ≥ 1, $P < 0.05$, at least 2 unique peptides, three biological replicates] and P-values were determined using Welch's ANOVA. Data is tabulated in **Supplementary Tables 16-18** and all annotations were exported as described above. **D,** Volcano plots for on-state EGFR neighborhoods that result after 60 min treatment with EGF versus no treatment using ICD ecto-tag MultiMap. After EGF treatment, cells were put on ice and illuminated to trigger labeling using either aryl-diazirine-biotin, aryl-azide-biotin, phenol-biotin photo-probes. Significantly enriched proteins are highlighted in the red box [log2(fold enrichment) ≥ 1, $P < 0.05$, at least 2 unique peptides, three biological replicates] and P-values were determined using Welch's ANOVA. Data is tabulated in **Supplementary Tables 19-21** and all annotations were exported as described above.

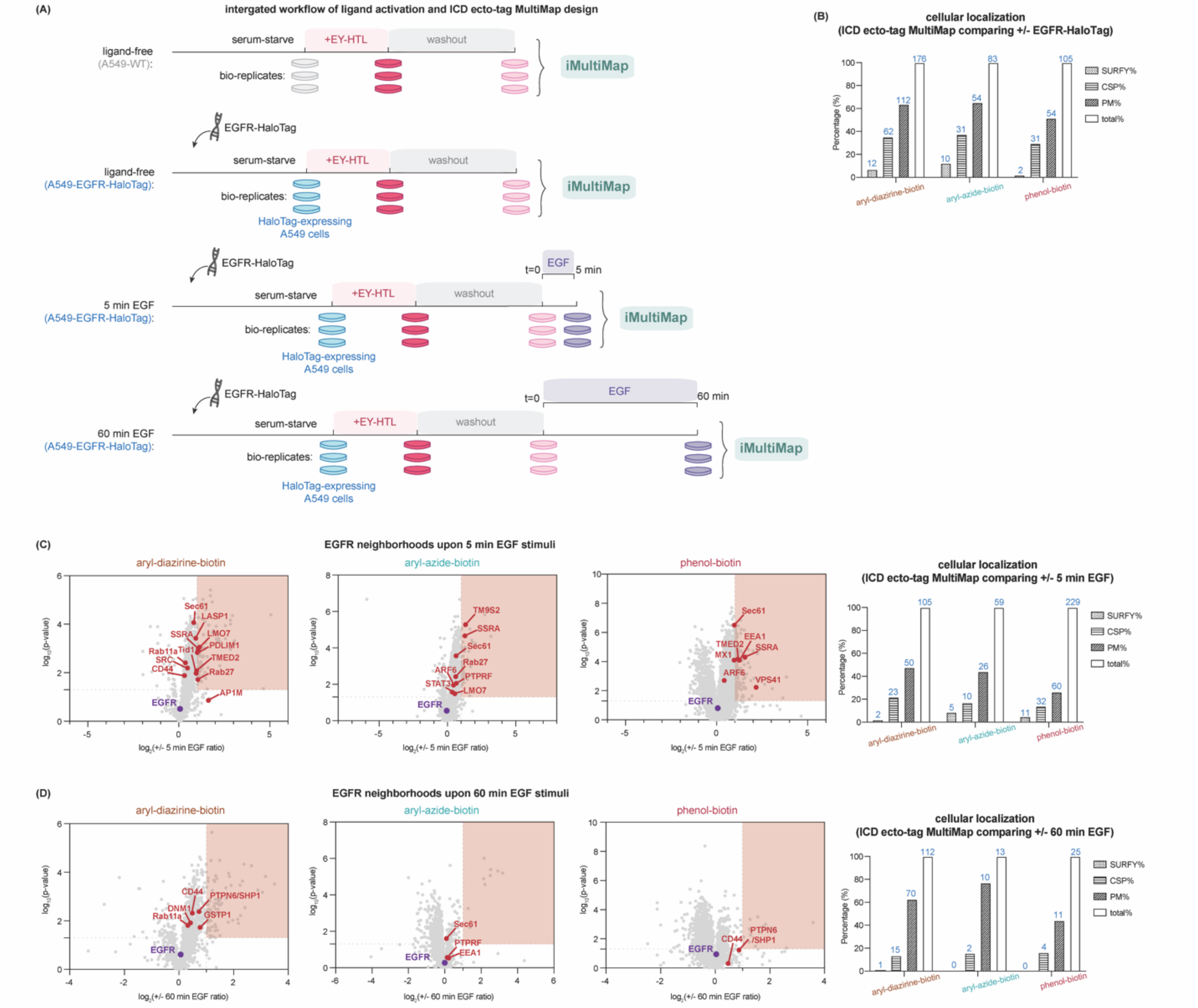

Supplementary Fig. 6 | Different and shared features identified via ECD and ICD ecto-tag MultiMap. A, Heatmap of the proteins identified in **Supplementary Figs. 5b-c**. Color indicates enrichment ratio of treatment over ligand-free conditions, with red indicating increased enrichment and blue indicating decreased enrichment. **B,** Venn diagram of enriched EGFR neighborhood via ICD ecto-tag MultiMap with 5 min incubation of EGF on A549 cells by using different photo-probes. **C,** Direct comparison of enriched EGFR neighborhood captured with 5 min or 60 min incubation of EGF. **D,** Gene Ontology (GO) analysis performed on all EGFR-interacting candidates from ICD ecto-tag MultiMap as described for **Supplementary Fig. 4e**. **E,** Venn diagram of enriched EGFR neighborhoods via ECD and ICD ecto-tag MultiMap at either 5 min or 60 min EGF activation showed significant difference between the two sets of datasets. **F,** Direct inter-comparison of our datasets with published EGFR and STS1 interactome datasets using well-established AP-MS and APEX2 methods in cells other than A549 cells.[3, 4]

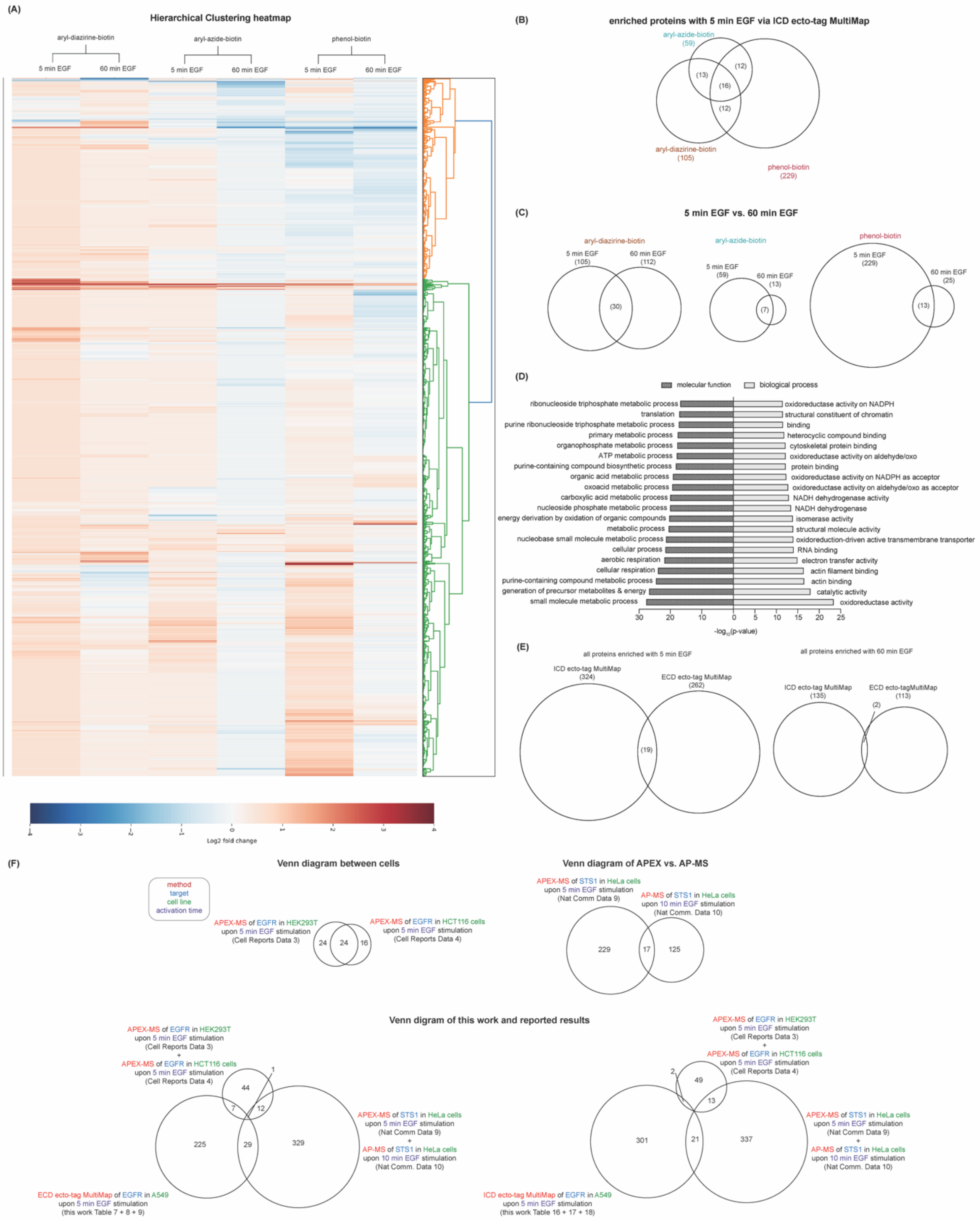
(A)
Hierarchical Clustering heatmap
aryl-diazirine-biotin
aryl-azide-biotin
phenol-biotin
5 min EGF
60 min EGF
Log2 fold change
(B)
enriched proteins with 5 min EGF via ICD ecto-tag MultiMap
aryl-azide-biotin (59)
aryl-diazirine-biotin (105)
phenol-biotin (229)
(13)
(12)
(16)
(12)
(C)
5 min EGF vs. 60 min EGF
aryl-diazirine-biotin
5 min EGF (105)
60 min EGF (112)
(30)
aryl-azide-biotin
5 min EGF (59)
60 min EGF (13)
(7)
phenol-biotin
5 min EGF (229)
60 min EGF (25)
(13)
(D)
molecular function
biological process
ribonucleoside triphosphate metabolic process
translation
purine ribonucleoside triphosphate metabolic process
primary metabolic process
organophosphate metabolic process
ATP metabolic process
purine-containing compound biosynthetic process
organic acid metabolic process
oxoacid metabolic process
carboxylic acid metabolic process
nucleoside phosphate metabolic process
energy derivation by oxidation of organic compounds
metabolic process
nucleobase small molecule metabolic process
cellular process
aerobic respiration
cellular respiration
purine-containing compound metabolic process
generation of precursor metabolites & energy
small molecule metabolic process
oxidoreductase activity on NADPH
structural constituent of chromatin
binding
heterocyclic compound binding
cytoskeletal protein binding
oxidoreductase activity on aldehyde/oxo
protein binding
oxidoreductase activity on NADPH as acceptor
oxidoreductase activity on aldehyde/oxo as acceptor
NADH dehydrogenase activity
NADH dehydrogenase
isomerase activity
structural molecule activity
oxidoreduction-driven active transmembrane transporter
RNA binding
electron transfer activity
actin filament binding
actin binding
catalytic activity
oxidoreductase activity
$-\log_{10}$(p-value)
(E)
all proteins enriched with 5 min EGF
ICD ecto-tag MultiMap (324)
ECD ecto-tag MultiMap (262)
(19)
all proteins enriched with 60 min EGF
ICD ecto-tag MultiMap (135)
ECD ecto-tagMultiMap (113)
(2)
(F)
Venn diagram between cells
method
target
cell line
activation time
APEX-MS of EGFR in HEK293T upon 5 min EGF stimulation (Cell Reports Data 3)
APEX-MS of EGFR in HCT116 cells upon 5 min EGF stimulation (Cell Reports Data 4)
24
24
16
Venn diagram of APEX vs. AP-MS
APEX-MS of STS1 in HeLa cells upon 5 min EGF stimulation (Nat Comm Data 9)
AP-MS of STS1 in HeLa cells upon 10 min EGF stimulation (Nat Comm. Data 10)
229
17
125
Venn digram of this work and reported results
APEX-MS of EGFR in HEK293T upon 5 min EGF stimulation (Cell Reports Data 3) + APEX-MS of EGFR in HCT116 cells upon 5 min EGF stimulation (Cell Reports Data 4)
APEX-MS of STS1 in HeLa cells upon 5 min EGF stimulation (Nat Comm Data 9) + AP-MS of STS1 in HeLa cells upon 10 min EGF stimulation (Nat Comm. Data 10)
ECD ecto-tag MultiMap of EGFR in A549 upon 5 min EGF stimulation (this work Table 7 + 8 + 9)
1
44
7
12
225
29
329
ICD ecto-tag MultiMap of EGFR in A549 upon 5 min EGF stimulation (this work Table 16 + 17 + 18)
2
49
13
301
21
337

Supplementary Fig. 7 | Genetic manipulation of target genes revealed EGFR ligand-dependent functional neighbors. A, Western blot showing siRNA knock-down of candidate functional neighbors identified in this study, including *SRC*, *ARF6*, *PTPRF*, *EPS15*, *RAB11A*, *DNM1* and *EGFR* in A549 cells. Normalized quantification results were shown beneath each band of interest. EGFR levels remain unchanged with neighbor gene knockdowns. **B,** Establishment of a time-course flow cytometry workflow to visualize cell surface EGFR in WT A549 cells. Cell surface EGFR levels were characterized at different timepoints of EGF stimulation. **C,** Representative flow cytometry of EGFR internalization assay monitoring cell surface EGFR upon EGF stimulation in cells with *EPS15*, *RAB11A*, *DNM1* or *ARF6* knocked down at different timepoints of EGF stimulation. **D,** Changes of EGFR level upon EGF stimulation and cycloheximide (100 ng/μL) to monitor EGFR degradation in cells with *EPS15*, *RAB11A, DNM1* or *ARF6* knocked down.

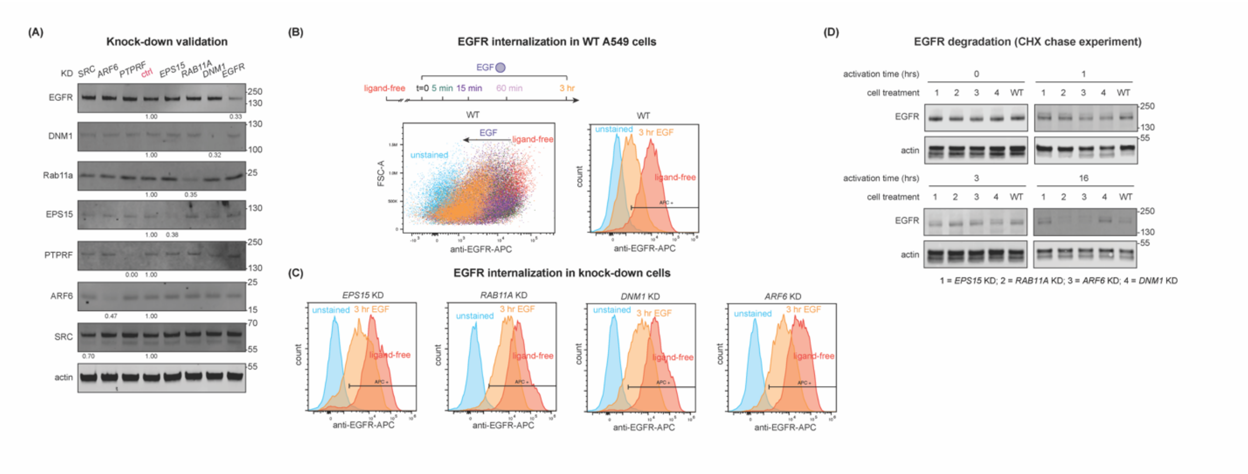

Supplementary Methods:

General chemical methods and instrumentation.

Chemicals were purchased including TFPA-PEG$_3$-biotin (Thermo Scientific, 21303), biotinyl tyramide (Sigma-Aldrich, SML2135) and Eosin Y (Sigma-Aldrich, E4009). DBCO-PEG$_3$-EY was synthesized and characterized by ChemPartner as previously reported. Aryl-diazirine-biotin (diazirine-PEG$_3$-biotin) was synthesized and characterized by Medicilon as previously reported. Eosin Y-HaloTag ligand was synthesized in house first before being batch synthesized and characterized by ChemPartner as shown in Scheme 1. All solvents and reagents were purchased from chemical suppliers (Sigma Aldrich, Acros Organics, Thermo Scientific or VWR Chemicals BDH®) and were used as received unless otherwise noted. Flash Column Chromatography was performed using Teledyne ISCO CombiFlash EZ Prep chromatography system, employing pre-packed silica gel Teledyne ISCO RediSep cartridges. Protein mass spectra were obtained using a Waters Xevo G2-XS time-of-flight mass spectrometer operating with Waters MassLynx software (version 4.2).

Complete list of antibodies and biological reagents.

Antibodies were purchased including: EGFR (Thermo Scientific, MA5-13319; Cell Signaling Technology, 4267, 2239, 2256), Rab11a (Cell Signaling Technology, 2413), STAT2 (Cell Signaling Technology, 72604), EPS15 (Cell Signaling Technology, 12460), SHP1 (Cell Signaling Technology, 26516), ITGA2 (Bethyl Laboratories, A305-211A-T; Cell Signaling Technology, 88228), Dynamin I/II (Cell Signaling Technology, 2342), SRC (Cell Signaling Technology, 2109), ARF6 (Cell Signaling Technology, 5740), PTPRF (Cell Signaling Technology, 61611), β-actin (Santa Cruz Biotechnology, sc-47778), CD44 (Cell Signaling Technology, 3578), Tid1 (Cell Signaling Technology, 4775), β-catenin (Cell Signaling Technology, 8480), GRB2 (Cell Signaling Technology, 3972), STAT3 (Cell Signaling Technology, 12640), Phospho-EGFR (Tyr1045) (Cell Signaling Technology, 2237), Phospho-EGFR (Tyr1086) (Cell Signaling Technology, 2220S), Phospho-EGFR (Tyr845) (Cell Signaling Technology, 2231), Phospho-EGFR (Tyr992) (Cell Signaling Technology 2235), Phospho-EGFR (Tyr1068) (Cell Signaling Technology, 3777), Phospho-EGFR (Tyr978) (Cell Signaling Technology, 3790), Phospho-EGFR (Tyr1173) (Cell Signaling Technology, 4757, 2244), Phospho-p44/42 MAPK (pERK) (Cell Signaling Technology, 4370, 9101), P44/42 MAPK (ERK1/2) (Cell Signaling Technology, 4695), Phospho-Akt (S473) (Cell Signaling Technology, 4058), Phospho-MEK (S217/221) (Cell Signaling Technology, 9154), Phospho-PLCy (Y783) (Cell Signaling Technology, 2821), Akt (Cell Signaling Technology, 4691), Phospho-EGFR (Y1068) Fluorescein-conjugated Antibody (R&D Systems, IC3570F), Phospho-EGFR Pathway Antibody panel (Cell Signaling Technology, 9789). Antibodies were used at 1:1000 dilution for Western blot, 1:2000 dilution for flow cytometry and 1:1000 dilution for imaging staining unless otherwise noted. In particular, Phospho-PLCy (Y783) (Cell Signaling Technology, 2821) was used at 1:500 dilution for Western blot. For PLA experiments, the following dilutions were used (Rab11a: 1:50; EPS15: 1:200; PTPRF: 1:800; SRC: 1:400; STAT3: 1:1000; ARF6: 1:50; DYN1: 1:30; CTNNB: 1:100; EGFR: 1:100)

The following antibodies were used in flow cytometry assays: streptavidin-AlexaFluor488 (Thermo Scientific, S32354), streptavidin-AlexaFluor647 (BioLegend, 405237), anti-EGFR-AlexaFluor647 (Fisher Scientific, 352918), anti-Flag-AlexaFluor647 (BioLegend, 637316). Other biological reagents such as recombinant Human EGF Protein (R&D Systems, 236-EG-200), Cetuximab (Selleck Chemicals, A2000), fluorescein-conjugated EGF (Thermo Scientific, E3478), Cycloheximide (Sigma-Aldrich, 01810-1G), flow cytometry fixation buffer (R&D Systems, FC004), Triton X-100 (1%) (Thermo Scientific, HFH10), Pierce™ High Capacity Streptavidin Agarose (Thermo Scientific, 20359), FITC Conjugation Kit (Abcam, ab188285), AlexaFluor 647 Conjugation Kit (Abcam, ab269823), Opti-MEM™ I Reduced Serum Medium (Thermo Scientific 31985062), TransIT-PRO® Transfection Reagent (Mirus Bio, MIR-5740), Lipofectamine

RNAiMAX Transfection Reagent (Thermo Scientific, 13778075), Glutathione (Selleck Chemicals, S4606), SOD1 protein (ACROBiosystems, SO1-H5148), Catalase (Sigma-Aldrich, C40-100), Glutathione peroxidase (Sigma-Aldrich, G6137-100) were purchased. Janelia Fluor-646 (JF646) with HaloTag ligand was a gift from the Lavis lab at Janelia.

For enrichment assays, Pierce™ Streptavidin Agarose beads (Pierce, 20349) and mini Bio-Spin columns (Bio-Rad, 732-6207) were used. Cell lysis buffer was prepared by diluting from 10X cell lysis buffer (Cell Signaling Technology, 9803S) or from 10X RIPA buffer (EMD Millipore, 20-188) and supplemented with protease inhibitor (Protease Inhibitor Cocktail 100X, Cell Signaling Technology, 5871) or phosphatase inhibitor (Phosphatase Inhibitor Cocktail 100X, Cell Signaling Technology, 5870) as indicated. Sample loading buffer was diluted from 4X Laemmli sample loading buffer (Bio-Rad, 161-0747). When performing solvent exchange processes, 7 kDa Zeba Spin desalting columns (ThermoFisher, 89883) were used.

Construct information and cell expression.
Plasmids for Flag-EGFR and Flag-EGFR-HaloTag were constructed in a pcDNA3.1(+) backbone by standard molecular biology methods as previously reported [5] using its native signal sequence with a GGGGS link between tags and EGFR sequence.

For gene knockout or knockdown, Cas9 constructs with sgRNA spacer sequences and commercial siRNAs (ThermoFisher Scientific, AM16708) were used as previously reported.[6] In short, spacer sequences listed below were cloned into the pCF822-recipient_U6-sgRNA-EFS-Cas9-P2A-Puro backbone (Addgene # 211645): Spacer sequences and their reverse complements were ordered with 5'CACC and 5'AAAC overhangs, then phosphorylated and annealed by incubating with T4 PNK (NEB M0201) (10 U) in T4 DNA Ligase Buffer (NEB B0202S) (10 µL, 1X). The pCF822-recipient_U6-sgRNA-EFS-Cas9-P2A-Puro backbone was digested with Esp3I (NEB R0734) (10 U) and dephosphorylated using CIP (NEB M0525) (6 U) for 2 hours at 37°C. The backbone was subsequently gel purified (Qiagen 28704). Backbone (50 ng) and phosphorylated spacers (0.5 pmol) were annealed with T4 DNA ligase (M0202) (400 U) for 2 hours at 25°C. The ligated plasmids were transformed into Mach1 competent cells (ThermoFisher Scientific, C862003). Plasmids were verified by whole plasmid sequencing after purification using either mini-prep (Qiagen 27104) or midi-prep kits (Zymo, D4200). All sequences were confirmed by Sanger (Quintarabio) and whole-plasmid (Primordium Labs) sequencing.

sgRNA spacer sequences	
Gene	Spacer sequence
SRC	TAGCAACAAGAGCAAGCCCA
SRC	GTCATAGAGGGCCACAAAGG
PTPRF (also known as *LAR*)	GTCGATGGAAGGGAACCCAG
PTPRF (also known as *LAR*)	GGGCACCATCGTCCTCCCTG
EPS15	TGATGGAAACACCATGGCTG
EPS15	TTAGCCGACACAGATGGCAA
RAB11A	TGTTGCAAACTCTACTCCAA
RAB11A	CCATGGCCTCACCTTTAAAG
EGFR	GATGTTCAATAACTGTGAGG
EGFR	AGCGATGCGACCCTCCGGGA
ARF6	GGTGACCACCATTCCCACTG

ARF6	TGTGGGTTTCAACGTGGAGA
DNM1	GGCCAGGTCAGAGTTGGCGG
DNM1	CATGGAAGATCTCATCCCGC
CD44	AAAGGACCCAGTCTTAGCTG
CD44	GAAAGGAGCAGCACTTCAGG
DNAJA3 (also known as *Tid1*)	TAAGGATGATCCCAAAGCCA
DNAJA3 (also known as *Tid1*)	TCAGGGAAGACGCAAAGGCG
PTPN6 (also known as *SHP1*)	GATGCAGAGACCCTGCTCAA
PTPN6 (also known as *SHP1*)	AAAGCACGAAGTCTCCAGGC
ITGA2	GCAACCATGACAATATACTG
ITGA2	TCAGATGGAAATGTCACAAG
ALCAM (also known as *CD166*)	GAGGCAGAAGAGCAGACGGC
ALCAM (also known as *CD166*)	CCTGGAGTACAAGACAACCA
PTPN11 (also known as *SHP2*)	GGAGGAACATGACATCGCGG
PTPN11 (also known as *SHP2*)	GTAGGATCTGCACAGTTCAG
RHEB	CCATATCCAACAATTTGCCA
RHEB	AGGGTGATCAGTTATGAAGA
TMED10	GAGGAGATGCGTGATACCAA
TMED10	AGATGGCAAGGACCAATCTG
SCARB1	GCTCTTCACGGTGTTCACGG
SCARB1	ACAGCCTCACCTGTACACGT
AP1M1	GCTGCGCAGGACATTGCCGT
AP1M1	GTGACGGTGGCTGGTGGCCG
PHB1 (also known as *PHB*)	CCTTAGACACATCTGACCTT
PHB1 (also known as *PHB*)	TTACCAAGGACACGTCATCC
STAT3	GAAGGCGTGATTCTTCCCAC
STAT3	GGAACAGATGCTCACTGCGC
CTNNB1 (also known as *CTNNB*)	GATGGAGTTGGACATGGCCA
CTNNB1 (also known as *CTNNB*)	GAAGCTGAACAAGAGTCCCA
CLPTM1	CCGGAGCGGGAAGATGGCGG
CLPTM1	TCAGCAGTTGGTTCCGCCGA

Supplementary Note 1:

Fig. 1B

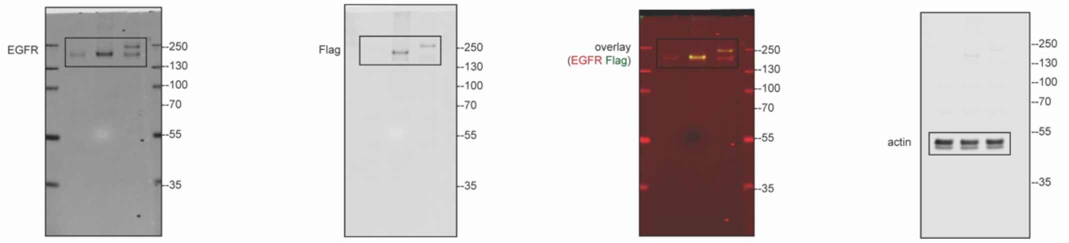

Fig. 2C

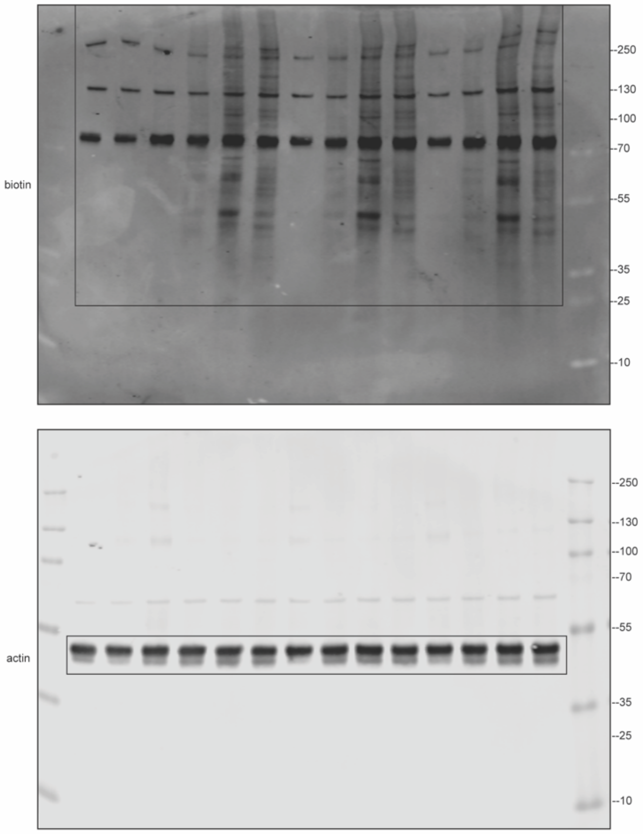

Fig. 3C

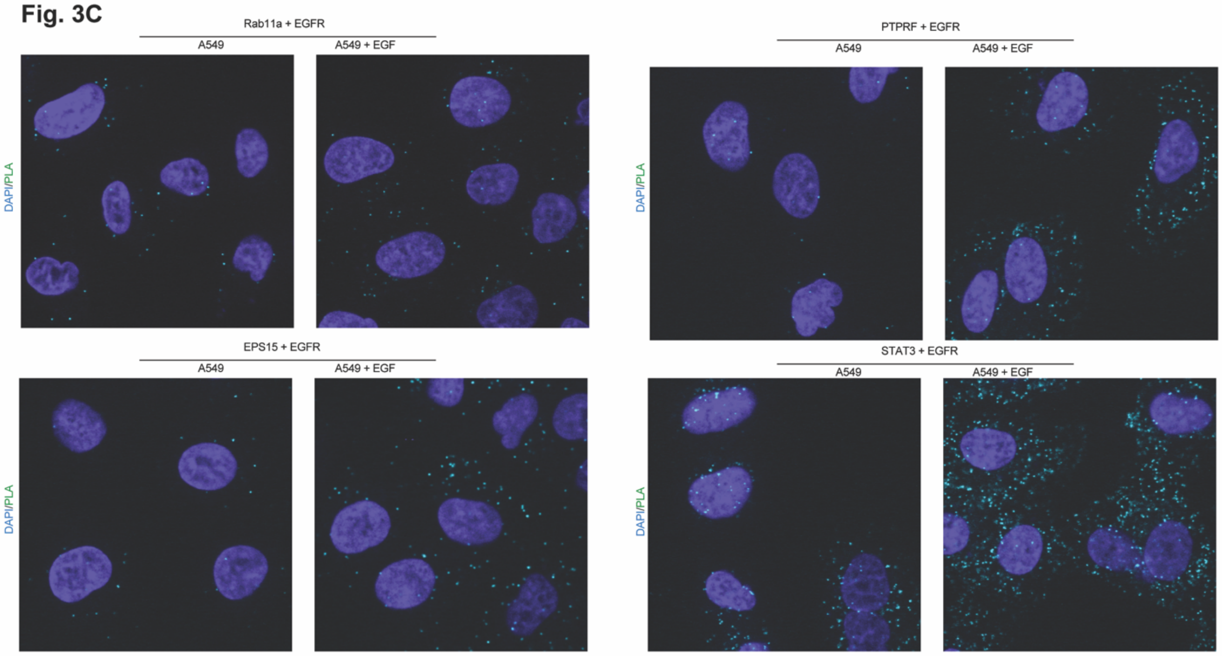

Rab11a + EGFR
A549
A549 + EGF
DAPI/PLA
PTPRF + EGFR
A549
A549 + EGF
DAPI/PLA
EPS15 + EGFR
A549
A549 + EGF
DAPI/PLA
STAT3 + EGFR
A549
A549 + EGF
DAPI/PLA

Fig. 4A

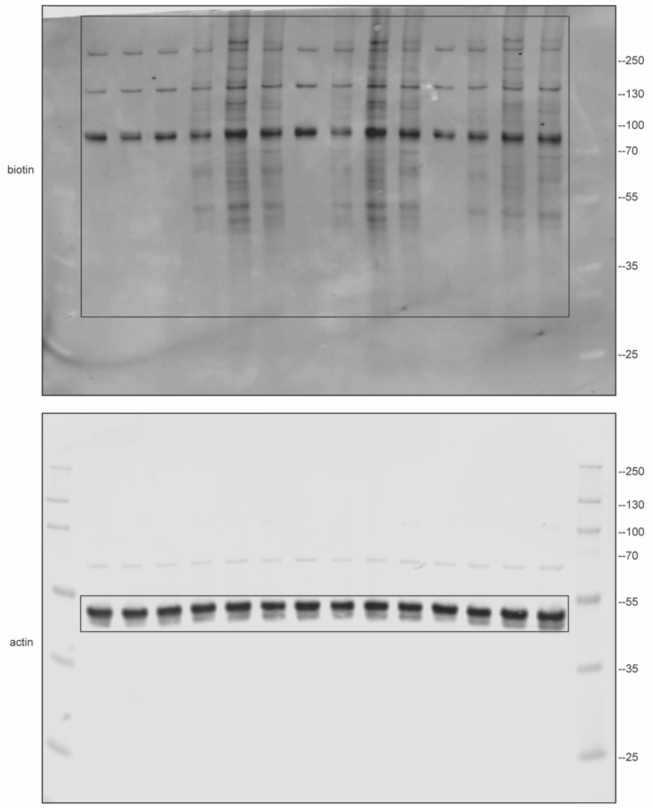

Fig. 4D

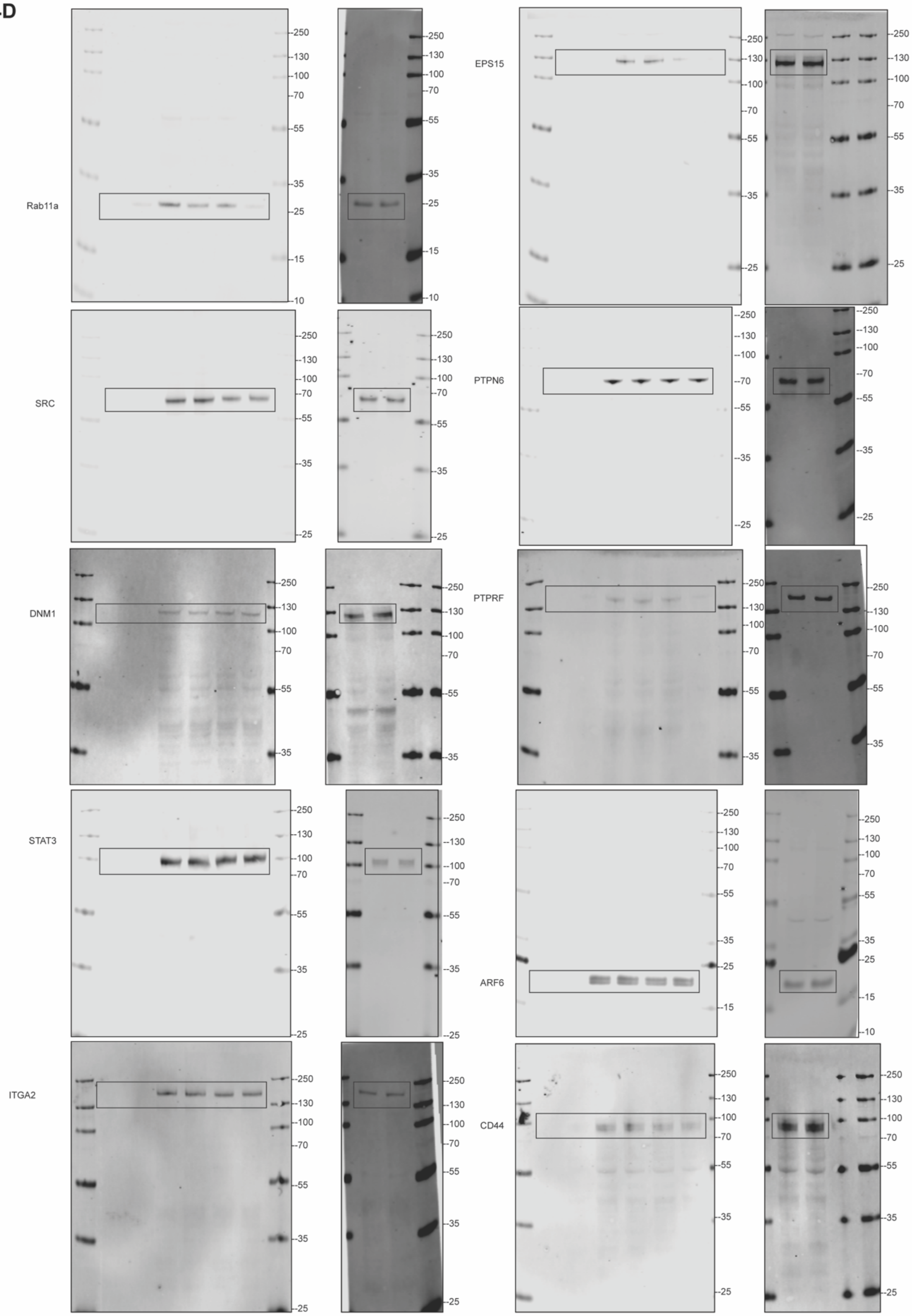

Rab11a
SRC
DNM1
STAT3
ITGA2
EPS15
PTPN6
PTPRF
ARF6
CD44
250
130
100
70
55
35
25
15
10

Fig. 5B

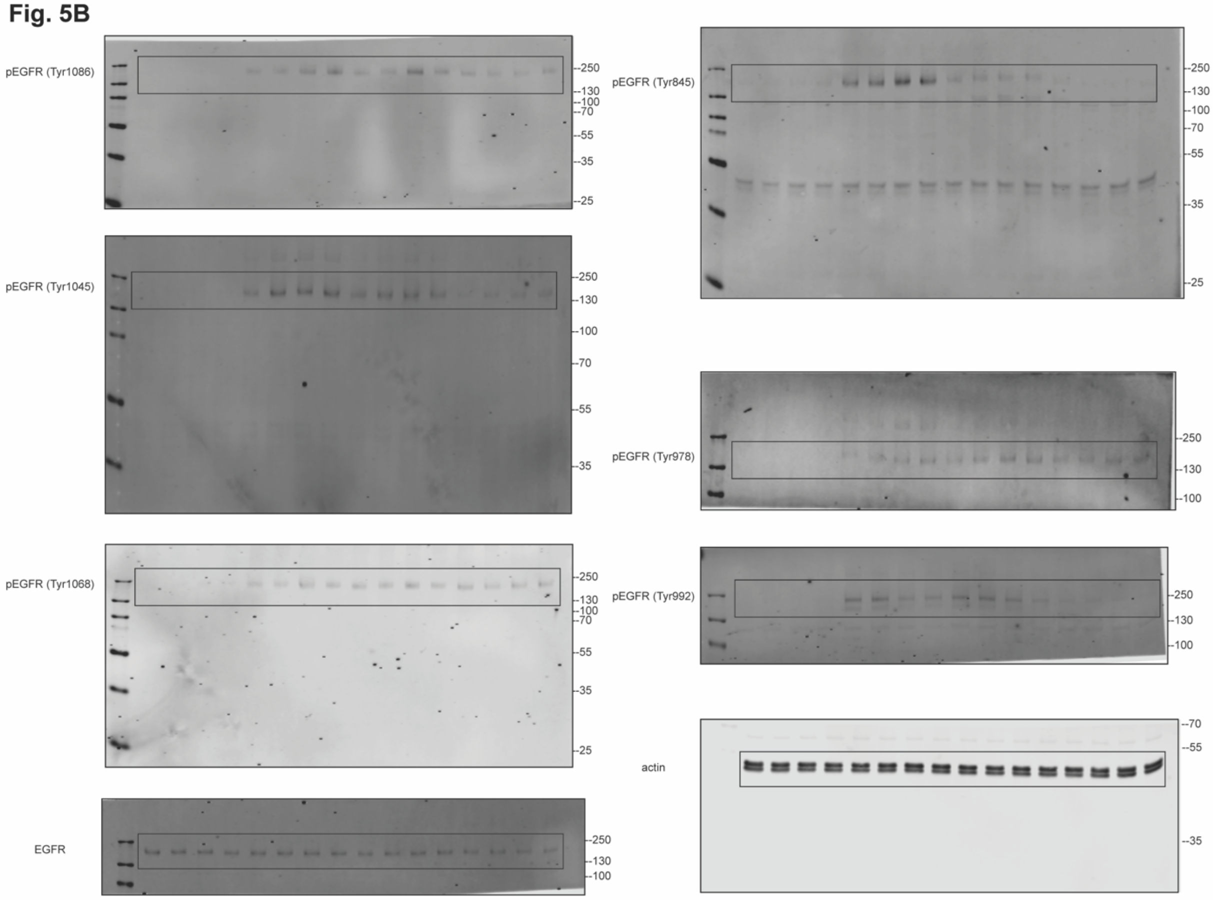
pEGFR (Tyr1086)
250
130
100
70
55
35
25
pEGFR (Tyr1045)
250
130
100
70
55
35
pEGFR (Tyr1068)
250
130
100
70
55
35
25
EGFR
250
130
100
pEGFR (Tyr845)
250
130
100
70
55
35
25
pEGFR (Tyr978)
250
130
100
pEGFR (Tyr992)
250
130
100
actin
70
55
35

Supplementary Fig. 1C

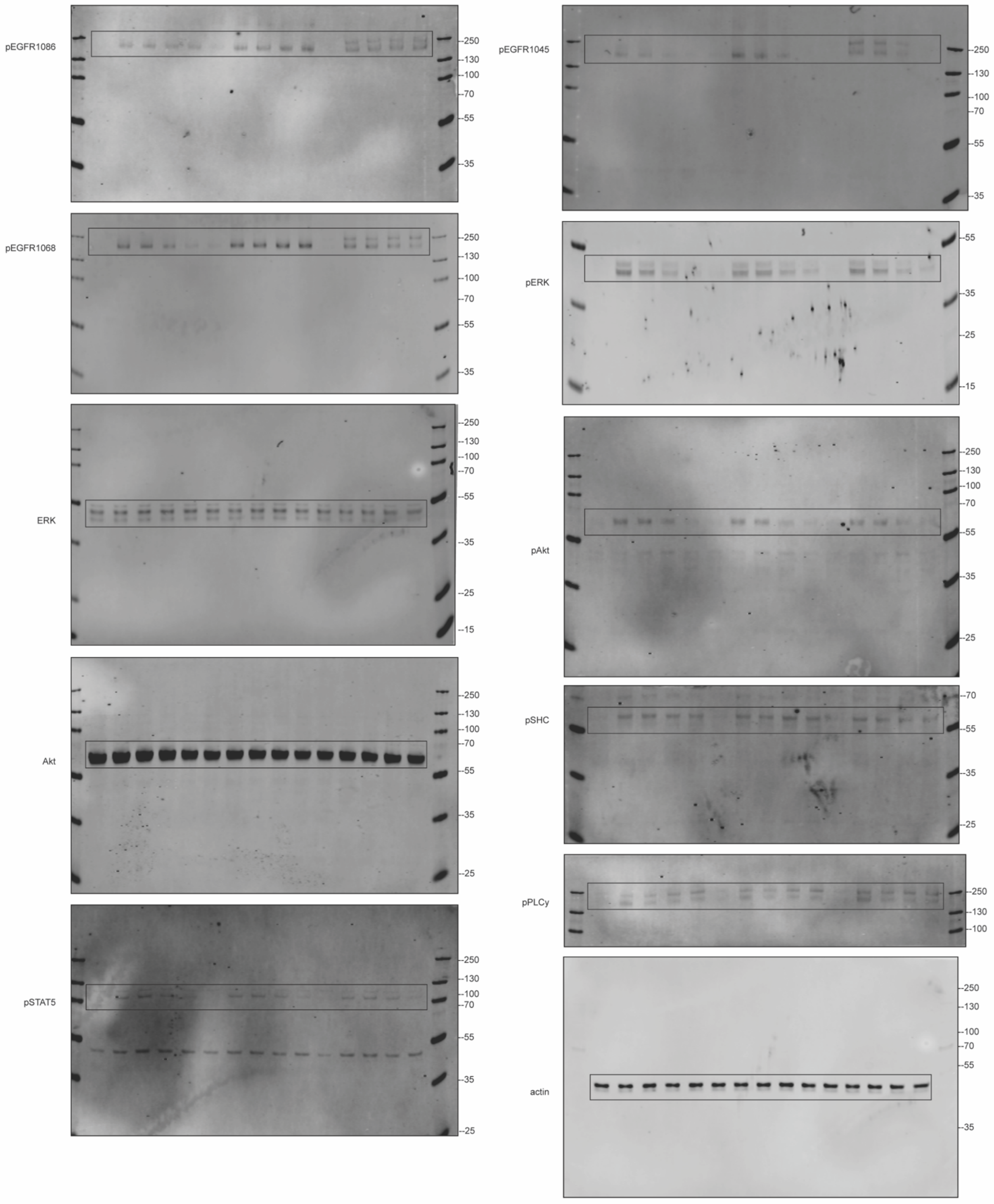

Supplementary Fig. 1F

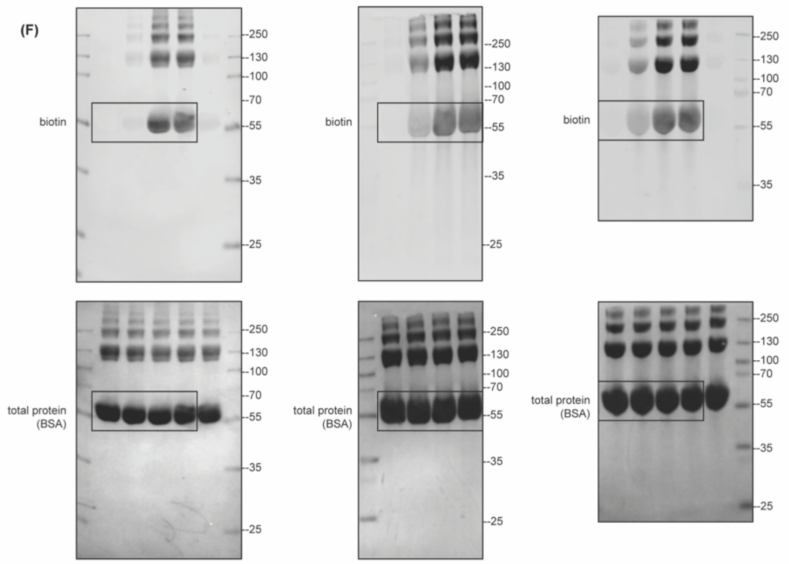

Supplementary Fig. 1G

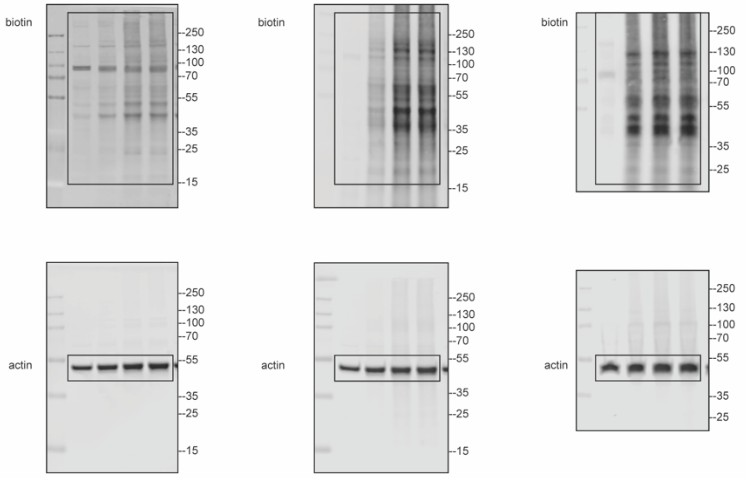

Supplementary Fig. 2E

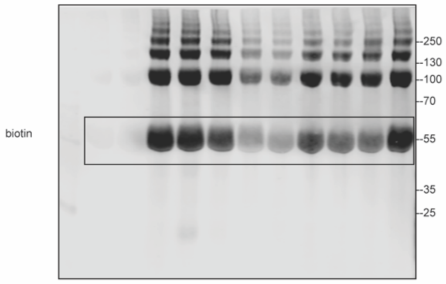

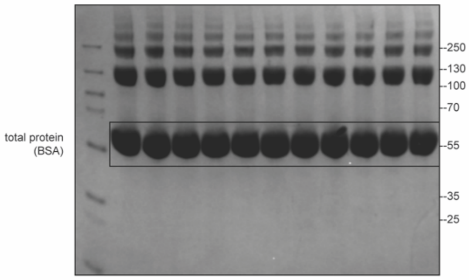

Supplementary Fig. 2F

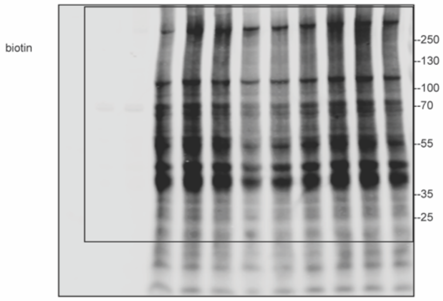

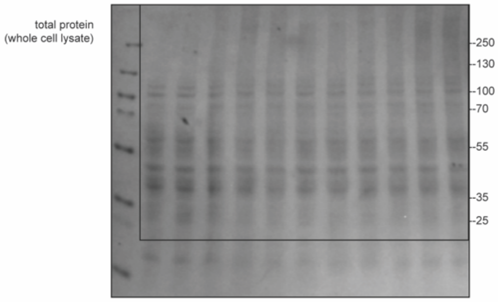

Supplementary Fig. 7A

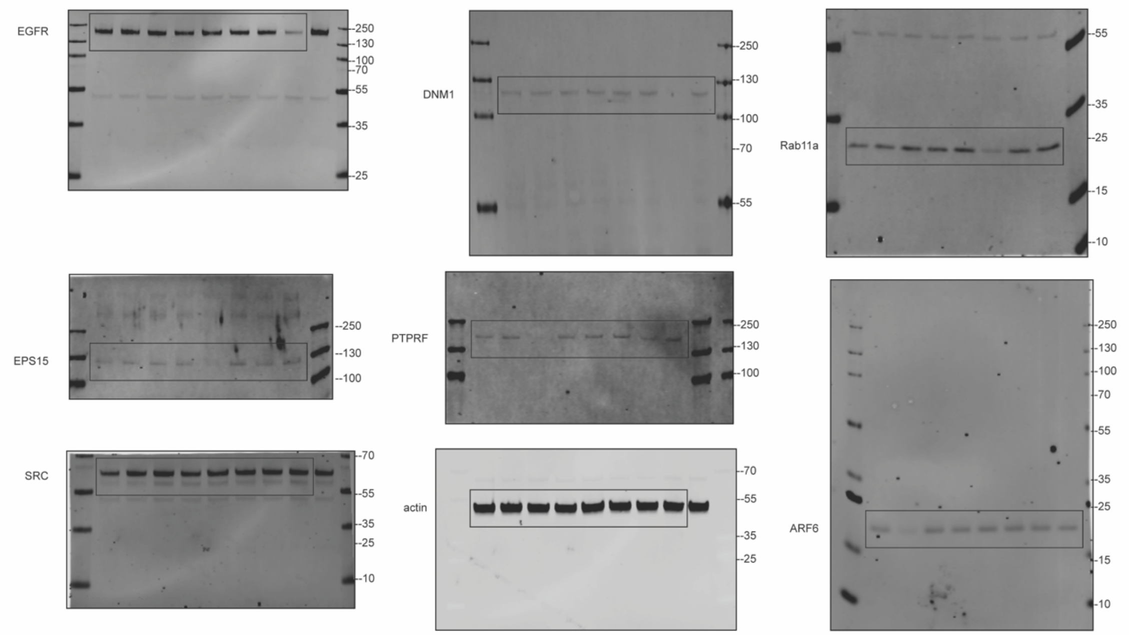

Supplementary Fig. 7D

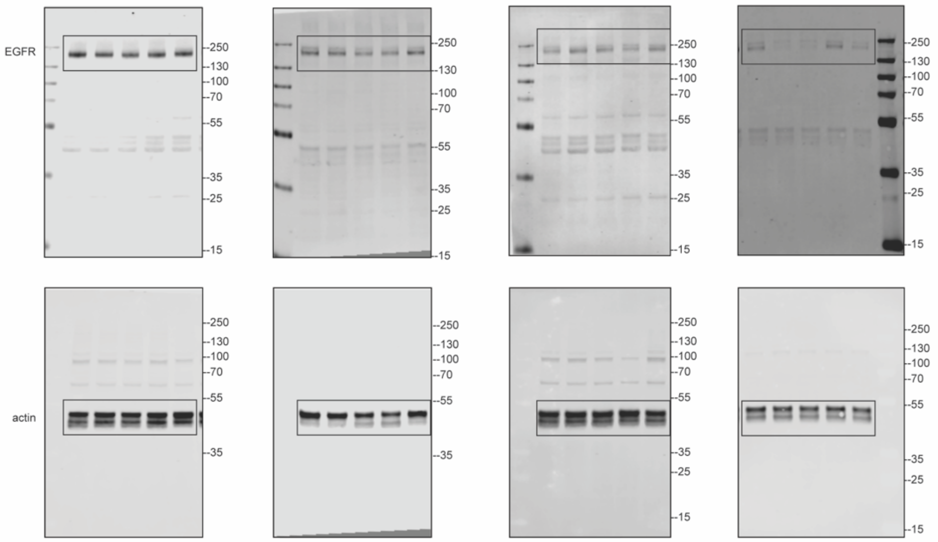

Supplementary Note 2:

Synthesis of EY-HaloTag Ligand (EY-HTL), 2',4',5',7'-tetrabromo-N-(2-(2-[(6-chlorohexyl)oxy]ethoxy)ethyl)-3',6'-dihydroxy-3-oxo-3H-spiro[2-benzofuran-1,9'-xanthene]-6-carboxamide.

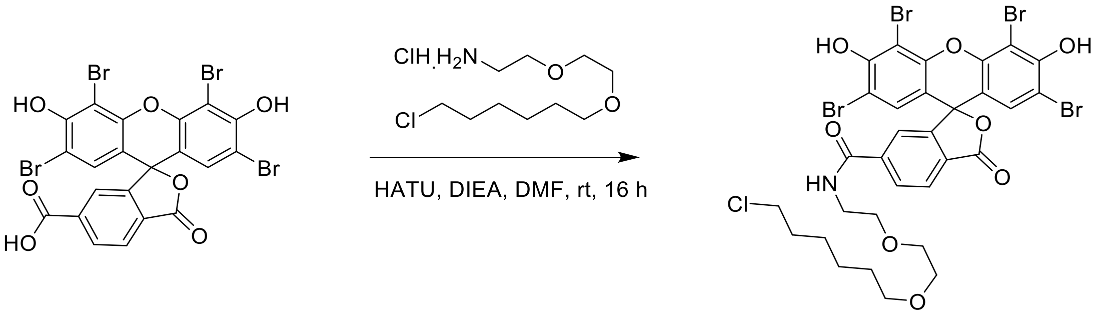

EY-HTL was synthesized similarly to reported literature.7 To a mixture of 2',4',5',7'-tetrabromo-3',6'-dihydroxy-3-oxo-3H-spiro[2-benzofuran-1,9'-xanthene]-6-carboxylic acid (50mg, 0.072mmol), DIPEA (28.02mg, 0.22mmol) and 1-(2-(2-aminoethoxy)ethoxy)-6-chlorohexane (20.68mg, 0.092mmol) in DMF (5mL) was added HATU (32.97mg, 0.087mmol) in portions at 0 ℃ under Ar atmosphere. The resulting mixture was stirred for 16 hours at room temperature under Ar atmosphere. The residue was purified by C18 column chromatography eluted with ACN: H2O (5% ~95%) to afford desired product (23mg, yield 35.46%) as a red solid. Synthesis and characterization were performed by Medicilon. LCMS: IDSUF06-WX-MS, Method: 95_15 min, t=6.515 min, MS (ESI): m/z=897.7 [M+H] +. 1H NMR (400 MHz, DMSO-d6): δ 10.83 (s, 1H), 8.76 (d, J = 46.5 Hz, 1H), 8.82-8.71 (m, 1H), 8.24-8.09 (m, 2H), 7.85-7.80 (m, 1H), 7.02 (s, 2H), 3.48-3.35 (m 10H), 3.30-3.27 (m, 2H), 1.67-1.63 (m, 2H), 1.41-1.23 (m, 6H).

Supplementary References:

1. Bausch-Fluck, D.; Goldmann, U.; Muller, S.; van Oostrum, M.; Muller, M.; Schubert, O. T.; Wollscheid, B., The in silico human surfaceome. *Proc Natl Acad Sci U S A* **2018,** *115* (46), E10988-E10997.
2. Li, Y.; Wang, Y.; Yao, Y.; Lyu, J.; Qiao, Q.; Mao, J.; Xu, Z.; Ye, M., Rapid Enzyme-Mediated Biotinylation for Cell Surface Proteome Profiling. *Anal Chem* **2021,** *93* (10), 4542-4551.
3. Ke, M.; Yuan, X.; He, A.; Yu, P.; Chen, W.; Shi, Y.; Hunter, T.; Zou, P.; Tian, R., Spatiotemporal profiling of cytosolic signaling complexes in living cells by selective proximity proteomics. *Nat Commun* **2021,** *12* (1), 71.
4. Perez Verdaguer, M.; Zhang, T.; Surve, S.; Paulo, J. A.; Wallace, C.; Watkins, S. C.; Gygi, S. P.; Sorkin, A., Time-resolved proximity labeling of protein networks associated with ligand-activated EGFR. *Cell Rep* **2022,** *39* (11), 110950.
5. Susa, K. J.; Rawson, S.; Kruse, A. C.; Blacklow, S. C., Cryo-EM structure of the B cell co-receptor CD19 bound to the tetraspanin CD81. *Science* **2021,** *371* (6526), 300-305.
6. Tan, I. L.; Perez, A. R.; Lew, R. J.; Sun, X.; Baldwin, A.; Zhu, Y. K.; Shah, M. M.; Berger, M. S.; Doudna, J. A.; Fellmann, C., Targeting the non-coding genome and temozolomide signature enables CRISPR-mediated glioma oncolysis. *Cell Rep* **2023,** *42* (11), 113339.
7. Takemoto, K.; Matsuda, T.; McDougall, M.; Klaubert, D. H.; Hasegawa, A.; Los, G. V.; Wood, K. V.; Miyawaki, A.; Nagai, T., Chromophore-assisted light inactivation of HaloTag fusion proteins labeled with eosin in living cells. *ACS Chem Biol* **2011,** *6* (5), 401-6.
