# Supplementary material for: Temporal photoproximity labeling of ligand-activated EGFR neighborhoods using MultiMap
Source: Nat Chem Biol. 2025 Nov 18;22(2):192–204. doi: 10.1038/s41589-025-02076-y (PMC12858401; doi:10.1038/s41589-025-02076-y)
Supplement: Supplementary file 2 — Reporting Summary [file 41589_2025_2076_MOESM2_ESM.pdf]

nature portfolio

Corresponding author(s): James A. Wells

Last updated by author(s): Sept 26, 2025

Reporting Summary

Nature Portfolio wishes to improve the reproducibility of the work that we publish. This form provides structure for consistency and transparency in reporting. For further information on Nature Portfolio policies, see our Editorial Policies and the Editorial Policy Checklist.

Statistics

For all statistical analyses, confirm that the following items are present in the figure legend, table legend, main text, or Methods section.

n/a	Confirmed	
☐	☒	The exact sample size (*n*) for each experimental group/condition, given as a discrete number and unit of measurement
☐	☒	A statement on whether measurements were taken from distinct samples or whether the same sample was measured repeatedly
☐	☒	The statistical test(s) used AND whether they are one- or two-sided<br>*Only common tests should be described solely by name; describe more complex techniques in the Methods section.*
☒	☐	A description of all covariates tested
☐	☒	A description of any assumptions or corrections, such as tests of normality and adjustment for multiple comparisons
☐	☒	A full description of the statistical parameters including central tendency (e.g. means) or other basic estimates (e.g. regression coefficient) AND variation (e.g. standard deviation) or associated estimates of uncertainty (e.g. confidence intervals)
☐	☒	For null hypothesis testing, the test statistic (e.g. *F*, *t*, *r*) with confidence intervals, effect sizes, degrees of freedom and *P* value noted<br>*Give P values as exact values whenever suitable.*
☒	☐	For Bayesian analysis, information on the choice of priors and Markov chain Monte Carlo settings
☒	☐	For hierarchical and complex designs, identification of the appropriate level for tests and full reporting of outcomes
☐	☒	Estimates of effect sizes (e.g. Cohen's *d*, Pearson's *r*), indicating how they were calculated

Our web collection on statistics for biologists contains articles on many of the points above.

Software and code

Policy information about availability of computer code

Data collection	Flow cytometry data were collected on a CytoFlex flow cytometer (Beckman CytoFlex). Protein quantification was performed on a multimode microplate reader Infinite 200 PRO (Tecan Trading AG, Switzerland). Immunoblots images were collected on an infrared LI-COR imager (Odyssey CLx). In-gel fluorescence and immunoblot fluorescence signals were detected on a BioRad imager (ChemiDoc XRS+ System). Proteomics experiments were performed on a TimsTOF PRO (Bruker) equipped with a CaptiveSpray source and a nanoElute system. Co-localization confocal microscopy was performed using a 60X Plan-Apo 679 1.42 NA oil objective on an Olympus Fluoview 3000 laser scanning confocal microscope. Other images were obtained on an inverted Nikon Ti-E microscope equipped with a Yokogawa CSU-22 confocal scanner unit.
Data analysis	Flow cytometry data were analyzed using FlowJo software (v10.6.1). WB analysis was performed using ImageStudioLite (v5.2.5). Proteomics data was searched using PEAKS online Xpro 1.6 (Bioinformatics Solutions Inc.) or FragPipe powered by MSFragger (v3.7). DNA and protein sequences were analyzed using SnapGene (v6.0.2). All other data was analyzed and visualized using GraphPad Prism (v8.0.1) and Microsoft Excel (v16.22).

For manuscripts utilizing custom algorithms or software that are central to the research but not yet described in published literature, software must be made available to editors and reviewers. We strongly encourage code deposition in a community repository (e.g. GitHub). See the Nature Portfolio guidelines for submitting code & software for further information.

April 2023

Data

Policy information about availability of data

All manuscripts must include a data availability statement. This statement should provide the following information, where applicable:

- Accession codes, unique identifiers, or web links for publicly available datasets
- A description of any restrictions on data availability
- For clinical datasets or third party data, please ensure that the statement adheres to our policy

All experimental data, materials and methods are available in the main text or the supplementary materials along with the source data. The MS proteomics data have been deposited to the ProteomeXchange Consortium through the PRIDE partner repository with the dataset identifier PXD059180.

Research involving human participants, their data, or biological material

Policy information about studies with human participants or human data. See also policy information about sex, gender (identity/presentation), and sexual orientation and race, ethnicity and racism.

Reporting on sex and gender	N/A
Reporting on race, ethnicity, or other socially relevant groupings	N/A
Population characteristics	N/A
Recruitment	N/A
Ethics oversight	N/A

Note that full information on the approval of the study protocol must also be provided in the manuscript.

Field-specific reporting

Please select the one below that is the best fit for your research. If you are not sure, read the appropriate sections before making your selection.

☒ Life sciences ☐ Behavioural & social sciences ☐ Ecological, evolutionary & environmental sciences

For a reference copy of the document with all sections, see nature.com/documents/nr-reporting-summary-flat.pdf

Life sciences study design

All studies must disclose on these points even when the disclosure is negative.

Sample size	Statistical methods to predetermine sample size were not conducted. Sample size for data collection was chosen to provide visualizable interpretation and statistical significance. For flow cytometry experiments, at least 10,000 events were counted. For proteomics analysis, biological triplicates for both samples and controls were evaluated as a commonly accepted standard for proteomics studies. For all other studies, sample sizes were determined based on standard practices.
Data exclusions	No data were excluded from analyses.
Replication	All experiments were performed in biological replicate or triplicate unless otherwise noted. We confirmed all replication were successful.
Randomization	Randomization was not applicable for this study since samples were allocated to experimental groups based on experimental design instead of random assignment.
Blinding	Blinding was not applicable for this study since all measurements were obtained using objective assays and readouts, thus eliminating potential investigator bias in data collection and analysis.

Reporting for specific materials, systems and methods

We require information from authors about some types of materials, experimental systems and methods used in many studies. Here, indicate whether each material, system or method listed is relevant to your study. If you are not sure if a list item applies to your research, read the appropriate section before selecting a response.

Materials & experimental systems

n/a	Involved in the study	
☐	☒	Antibodies
☐	☒	Eukaryotic cell lines
☒	☐	Palaeontology and archaeology
☒	☐	Animals and other organisms
☒	☐	Clinical data
☒	☐	Dual use research of concern
☒	☐	Plants

Methods

n/a	Involved in the study	
☒	☐	ChIP-seq
☐	☒	Flow cytometry
☒	☐	MRI-based neuroimaging

Antibodies

Antibodies used

Antibodies were purchased including: EGFR (Thermo Scientific, MA5-13319; Cell Signaling Technology, 4267, 2239, 2256), Rab11a (Cell Signaling Technology, 2413), STAT2 (Cell Signaling Technology, 72604), EPS15 (Cell Signaling Technology, 12460), SHP1 (Cell Signaling Technology, 26516), ITGA2 (Bethyl Laboratories, A305-211A-T; Cell Signaling Technology, 88228), Dynamin I/II (Cell Signaling Technology, 2342), SRC (Cell Signaling Technology, 2109), ARF6 (Cell Signaling Technology, 5740), PTPRF (Cell Signaling Technology, 61611), β-actin (Santa Cruz Biotechnology, sc-47778), CD44 (Cell Signaling Technology, 3578), Tid1 (Cell Signaling Technology, 4775), β-catenin (Cell Signaling Technology, 8480), GRB2 (Cell Signaling Technology, 3972), STAT3 (Cell Signaling Technology, 12640), Phospho-EGFR (Tyr1045) (Cell Signaling Technology, 2237), Phospho-EGFR (Tyr1086) (Cell Signaling Technology, 2220S), Phospho-EGFR (Tyr845) (Cell Signaling Technology, 2231), Phospho-EGFR (Tyr992) (Cell Signaling Technology 2235), Phospho-EGFR (Tyr1068) (Cell Signaling Technology, 3777), Phospho-EGFR (Tyr978) (Cell Signaling Technology, 3790), Phospho-EGFR (Tyr1173) (Cell Signaling Technology, 4757, 2244), Phospho-p44/42 MAPK (pERK) (Cell Signaling Technology, 4370, 9101), P44/42 MAPK (ERK1/2) (Cell Signaling Technology, 4695), Phospho-Akt (S473) (Cell Signaling Technology, 4058), Phospho-MEK (S217/221) (Cell Signaling Technology, 9154), Phospho-PLCγ (Y783) (Cell Signaling Technology, 2821), Akt (Cell Signaling Technology, 4691), Phospho-EGFR (Y1068) Fluorescein-conjugated Antibody (R&D Systems, IC3570F), Phospho-EGFR Pathway Antibody panel (Cell Signaling Technology, 9789). Anti-M1-FLAG monoclonal antibody was purified from M1 hybridoma. The following antibodies were used in flow cytometry assays: streptavidin-AlexaFluor488 (Thermo Scientific, S32354), streptavidin-AlexaFluor647 (BioLegend, 405237), anti-EGFR-AlexaFluor647 (Fisher Scientific, 352918), anti-Flag-AlexaFluor647 (BioLegend, 637316). Secondary antibodies included anti-rabbit IgG Goat IR800 secondary antibody (Rockland, 926-32211), anti-rabbit IgG Goat IR680 secondary antibody (Rockland, 611-144-002), anti-rabbit IgG Goat secondary antibody peroxidase (Rockland, 611-1302), anti-mouse IgG Goat IR800 secondary antibody (Rockland, 610-145-211) and/or anti-mouse IgG Goat IR680 secondary antibody (Rockland, 610-144-002). Antibodies were used at 1:1000 dilution for Western blot, 1:2000 dilution for flow cytometry and 1:1000 dilution for imaging staining unless otherwise noted. In particular, Phospho-PLCγ (Y783) (Cell Signaling Technology, 2821) was used at 1:500 dilution for Western blot. For PLA experiments, the following dilutions were used (Rab11a: 1:50; EPS15: 1:200; PTPRF: 1:800; SRC: 1:400; STAT3: 1:1000; ARF6: 1:50; DYN1: 1:30; CTNNB: 1:100; EGFR: 1:100).

Validation

ll antibodies were validated by commercial manufacturer and provided on the manufacturer's websites.
EGFR (Thermo Scientific, MA5-13319) https://www.thermofisher.com/antibody/product/EGFR-Antibody-clone-199-12-Monoclonal/MA5-13319
EGFR (Cell Signaling Technology, 4267) https://www.cellsignal.com/products/primary-antibodies/egf-receptor-d38b1-xp-rabbit-mab/4267?srsltid=AfmBOoo78mL0Knnzfr7dYEO9m3g_wBy1RHG63L3Tbxxnx1h1crRYnjc7
EGFR (Cell Signaling Technology, 2239) https://www.cellsignal.com/products/primary-antibodies/egf-receptor-1f4-mouse-mab/2239
EGFR (Cell Signaling Technology, 2256) https://www.cellsignal.com/products/primary-antibodies/egf-receptor-egfr1-mouse-mab/2256
Rab11a (Cell Signaling Technology, 2413) https://www.cellsignal.com/products/primary-antibodies/rab11a-antibody/2413
STAT2 (Cell Signaling Technology, 72604) https://www.cellsignal.com/products/primary-antibodies/stat2-d9j7l-rabbit-mab/72604
EPS15 (Cell Signaling Technology, 12460) https://www.cellsignal.com/products/primary-antibodies/eps15-d3k8r-rabbit-mab/12460
SHP1 (Cell Signaling Technology, 26516) https://www.cellsignal.com/products/primary-antibodies/shp-1-e1u6r-rabbit-mab/26516
ITGA2 (Bethyl Laboratories, A305-211A-T) https://www.thermofisher.com/antibody/product/ITGA2-CD49B-Integrin-alpha-2-Antibody-Polyclonal/A305-211A
ITGA2 (Cell Signaling Technology, 88228) https://www.cellsignal.com/products/primary-antibodies/integrin-a2-antibody/88228
Dynamin I/II (Cell Signaling Technology, 2342) https://www.cellsignal.com/products/primary-antibodies/dynamin-1-2-antibody/2342
SRC (Cell Signaling Technology, 2109) https://www.cellsignal.com/products/primary-antibodies/src-36d10-rabbit-mab/2109
ARF6 (Cell Signaling Technology, 5740) https://www.cellsignal.com/products/primary-antibodies/arf6-d12g6-rabbit-mab/5740
PTPRF (Cell Signaling Technology, 61611) https://www.cellsignal.com/products/primary-antibodies/ptprf-lar-e6w4x-rabbit-mab/61611
β-actin (Santa Cruz Biotechnology, sc-47778) https://www.scbt.com/p/beta-actin-antibody-c4?srsltid=AfmBOopS71VTvFPu8B95myYNvIRyv72r664ENKmRv9v5jE7O5KeRVRCB
CD44 (Cell Signaling Technology, 3578) https://www.cellsignal.com/products/primary-antibodies/cd44-antibody/3578
Tid1 (Cell Signaling Technology, 4775) https://www.cellsignal.com/products/primary-antibodies/tid-1-rs13-mouse-mab/4775
β-catenin (Cell Signaling Technology, 8480) https://www.cellsignal.com/products/primary-antibodies/b-catenin-d10a8-xp-rabbit-mab/8480
GRB2 (Cell Signaling Technology, 3972) https://www.cellsignal.com/products/primary-antibodies/grb2-antibody/3972
STAT3 (Cell Signaling Technology, 12640) https://www.cellsignal.com/products/primary-antibodies/stat3-d3z2g-rabbit-mab/12640
Phospho-EGFR (Tyr1045) (Cell Signaling Technology, 2237) https://www.cellsignal.com/products/primary-antibodies/phospho-egf-receptor-tyr1045-antibody/2237
Phospho-EGFR (Tyr1086) (Cell Signaling Technology, 2220) https://www.cellsignal.com/products/primary-antibodies/phospho-egf-receptor-tyr1086-antibody/2220
Phospho-EGFR (Tyr845) (Cell Signaling Technology, 2231) https://www.cellsignal.com/products/primary-antibodies/phospho-egf-receptor-tyr845-antibody/2231

Phospho-EGFR (Tyr992) (Cell Signaling Technology 2235) https://www.cellsignal.com/products/primary-antibodies/phospho-egf-receptor-tyr992-antibody/2235
Phospho-EGFR (Tyr1068) (Cell Signaling Technology, 3777) https://www.cellsignal.com/products/primary-antibodies/phospho-egf-receptor-tyr1068-d7a5-xp-rabbit-mab/3777
Phospho-EGFR (Tyr978) (Cell Signaling Technology, 3790) https://www.cellsignal.com/products/primary-antibodies/phospho-her4-erbb4-tyr984-egfr-tyr978-antibody/3790
Phospho-EGFR (Tyr1173) (Cell Signaling Technology, 4757) https://www.cellsignal.com/products/primary-antibodies/phospho-her4-erbb4-tyr1284-egfr-tyr1173-21a9-rabbit-mab/4757
Phospho-EGFR (Tyr1173) (Cell Signaling Technology, 2244) https://www.cellsignal.com/products/primary-antibodies/phospho-her2-erbb2-tyr1248-egfr-tyr1173-antibody/2244
Phospho-p44/42 MAPK (pERK) (Cell Signaling Technology, 4370) https://www.cellsignal.com/products/primary-antibodies/phospho-p44-42-mapk-erk1-2-thr202-tyr204-d13-14-4e-xp-rabbit-mab/4370
Phospho-p44/42 MAPK (pERK) (Cell Signaling Technology, 9101) https://www.cellsignal.com/products/primary-antibodies/phospho-p44-42-mapk-erk1-2-thr202-tyr204-antibody/9101
P44/42 MAPK (ERK1/2) (Cell Signaling Technology, 4695) https://www.cellsignal.com/products/primary-antibodies/p44-42-mapk-erk1-2-137f5-rabbit-mab/4695
Phospho-Akt (S473) (Cell Signaling Technology, 4058) https://www.cellsignal.com/products/primary-antibodies/phospho-akt-ser473-193h12-rabbit-mab/4058
Phospho-MEK (S217/221) (Cell Signaling Technology, 9154) https://www.cellsignal.com/products/primary-antibodies/phospho-mek1-2-ser217-221-41g9-rabbit-mab/9154
Phospho-PLCy (Y783) (Cell Signaling Technology, 2821) https://www.cellsignal.com/products/primary-antibodies/phospho-plcg1-tyr783-antibody/2821
Akt (Cell Signaling Technology, 4691) https://www.cellsignal.com/products/primary-antibodies/akt-pan-c67e7-rabbit-mab/4691
Phospho-EGFR (Y1068) Fluorescein-conjugated Antibody (R&D Systems, IC3570F) https://www.rndsystems.com/products/phospho-egfr-y1068-fluorescein-conjugated-antibody-338324_ic3570f
Phospho-EGFR Pathway Antibody panel (Cell Signaling Technology, 9789) https://www.cellsignal.com/products/primary-antibodies/phospho-egf-receptor-pathway-antibody-sampler-kit/9789
streptavidin-AlexaFluor488 (Thermo Scientific, S32354) https://www.thermofisher.com/order/catalog/product/S32354
streptavidin-AlexaFluor647 (BioLegend, 405237) https://www.biolegend.com/en-gb/products/alexa-fluor-647-streptavidin-9305
anti-EGFR-AlexaFluor647 (Fisher Scientific, 352918) https://www.biolegend.com/ja-jp/products/alexa-fluor-647-anti-human-egfr-antibody-14302
anti-Flag-AlexaFluor647 (BioLegend, 637316) https://www.biolegend.com/fr-ch/products/alexa-fluor-647-anti-dykddddk-tag-14979
anti-rabbit IgG Goat IR800 secondary antibody (Rockland, 926-32211) https://www.rockland.com/categories/secondary-antibodies/rabbit-igg-hl-antibody-dylight-800-conjugated-611-145-002/?srsltid=AfmBOooshQ5DZkLvM1R1zvDeMcO-stCUC1fpwbs-btGTn9VT181QRZkq
anti-rabbit IgG Goat IR680 secondary antibody (Rockland, 611-144-002) https://www.rockland.com/categories/secondary-antibodies/rabbit-igg-hl-antibody-dylight-680-conjugated-611-144-002/?srsltid=AfmBOop2JFjBmX25RFO1MsxkIvduq9sg7DrSgGNdR9weWy9q5qBJAC8p
anti-rabbit IgG Goat secondary antibody peroxidase (Rockland, 611-1302) https://www.rockland.com/categories/secondary-antibodies/rabbit-igg-hl-secondary-antibody-peroxidase-conjugated-611-1302/
anti-mouse IgG Goat IR800 secondary antibody (Rockland, 610-145-121) https://www.rockland.com/categories/secondary-antibodies/mouse-igg-hl-antibody-dylight-800-conjugated-pre-adsorbed-610-145-121/?srsltid=AfmBOoqvGhSUURnvvp7e3PZHib5Hc4HWh4QDaHAbgW7ol2OhY4SKSf1o
anti-mouse IgG Goat IR680 secondary antibody (Rockland, 610-144-002) https://www.rockland.com/categories/secondary-antibodies/mouse-igg-hl-antibody-dylight-680-conjugated-610-144-002/

Eukaryotic cell lines

Policy information about cell lines and Sex and Gender in Research

Cell line source(s)	A549 cells, UCSF Cell and Genome Engineering Core (CGEC)
Authentication	Cell authentication was performed using short tandem repeat (STR) markers by UCSF Cell culture and banking services.
Mycoplasma contamination	Mycoplasma contamination tests were performed regularly with negative results.
Commonly misidentified lines (See ICLAC register)	None

Plants

Seed stocks	N/A
Novel plant genotypes	N/A
Authentication	N/A

Flow Cytometry

Plots

Confirm that:

☒ The axis labels state the marker and fluorochrome used (e.g. CD4-FITC).

☒ The axis scales are clearly visible. Include numbers along axes only for bottom left plot of group (a 'group' is an analysis of identical markers).

☐ All plots are contour plots with outliers or pseudocolor plots.

☐ A numerical value for number of cells or percentage (with statistics) is provided.

Methodology

Sample preparation	Cultured cells were incubated at 37 °C in 5% CO2 for the duration of the assay. To guarantee minimal cell activity, cells were moved onto ice right after treatment and washed three times with pre-chilled PBS. Whole cell flow staining or cell surface staining were then performed on pelleted cells. For cell surface staining, cells were blocked with filtered 3% BSA in PBS for 30 minutes at 4 °C and stained with corresponding antibody for 1 h at 4 °C. Stained cells were then washed three times with PBS and resuspended in PBS for flow cytometry analysis. For whole cell staining, cells were fixed with 4% formaldehyde for 10 min at room temperature, washed twice with PBS and then permeabilized with 100% pre-chilled methanol for 15 min at 4 °C. Cells were then washed before blocking with filtered 3% BSA in PBS for 30 minutes at 4 °C and stained with corresponding antibody for 1 h at 4 °C. Stained cells were then washed three times with PBS and resuspended in PBS for flow cytometry analysis.
Instrument	All experiments were performed on a CytoFlex flow cytometer (Beckman CytoFlex).
Software	FlowJo (v10.6.1)
Cell population abundance	More than 10,000 events were recorded.
Gating strategy	Live vs. dead cells were gated first using FSC-SSC gating. Single cells were then gated by removing doublets in FSC-H-FSC-A gating.

☐ Tick this box to confirm that a figure exemplifying the gating strategy is provided in the Supplementary Information.
